# Disentangling the Pitfalls of Rotating Disk Electrode-Based OER Stability Assessment: Bubble Blockage or Substrate Passivation?

**DOI:** 10.1021/acscatal.4c05447

**Published:** 2024-11-13

**Authors:** Aline Bornet, Pavel Moreno-García, Abhijit Dutta, Ying Kong, Mike Liechti, Soma Vesztergom, Matthias Arenz, Peter Broekmann

**Affiliations:** † Department of Chemistry, Biochemistry and Pharmaceutical Sciences, University of Bern, Freiestrasse 3, Bern 3012, Switzerland; ‡ MTA-ELTE Momentum Interfacial Electrochemistry Research Group, Eötvös Loránd University, Pázmány Péter sétány 1/A, Budapest 1117, Hungary

**Keywords:** oxygen evolution reaction, inverted rotating disk electrode, aqueous model systems, electrocatalyst stability, electrocatalysis

## Abstract

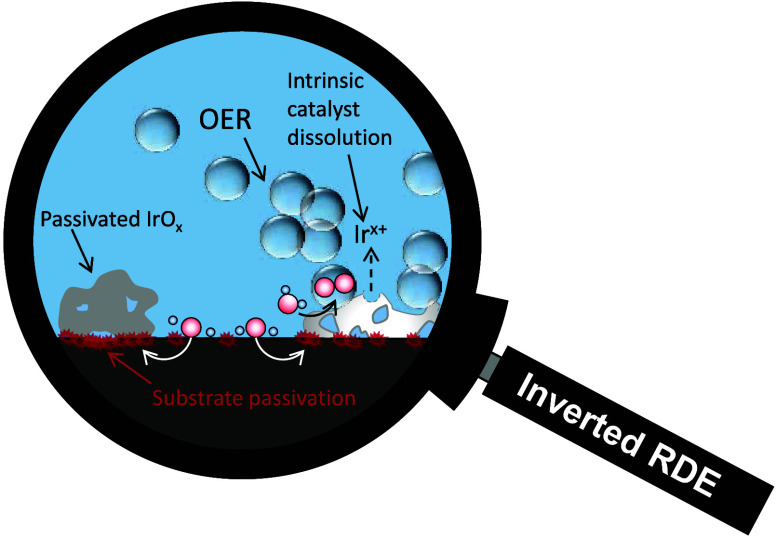

Oxygen evolution reaction (OER) catalyst stability metrics derived from aqueous model systems (AMSs) prove valuable only if they are transferable to technical membrane electrode assembly (MEA) settings. Currently, there is consensus that stability data derived from ubiquitous rotating disk electrode (RDE)-based investigations substantially overestimate material degradation mainly due to the nonideal inertness of catalyst-backing electrode materials as well as bubble shielding of the catalyst by evolved oxygen. Despite the independently developed understanding of these two processes, their interplay and relative impact on intrinsic and operational material stability have not yet been established. Herein, we employ an inverted RDE-based approach coupled with online gas chromatographic quantification that exploits buoyancy and anode hydrophilicity existing under operating acidic OER conditions and excludes the influence of bubble retention on the surface of the catalyst. This approach thus allows us to dissect the degradation process occurring during the RDE-based OER stability studies. We demonstrate that the stability discrepancy between galvanostatic nanoparticle (NP)-based RDE and MEA data does not originate from the accumulation of bubbles in the catalyst layer during water oxidation but from the utilization of corrosion-prone substrate materials in the AMS. Moreover, we provide mechanistic insights into the degradation process and devise experimental measures to mitigate or circumvent RDE-related limitations when the technique is to be applied to an OER catalyst stability assessment. These findings should facilitate the transferability between AMS and MEA approaches and promote further development of the latter.

## Introduction

Electrocatalytic gas-evolving reactions are of paramount relevance for energy-related electrochemical processes.^1−3^ Among them, the oxygen evolution reaction (OER) is the most common anodic reaction that is coupled to the cathodic production of H_2_ and other valuable products.^4^ Its understanding and optimized operation are the most needed prerequisites for the technical deployment of proton exchange membrane water electrolyzers (PEMWEs) that are expected to become the main source of future green hydrogen production.^5^ While assessment and optimization of OER catalyst activity has reached maturity, the benchmarking of catalyst stability is not yet sufficiently advanced and is the subject of current research.^6−9^ Typically, state-of-the-art Ir-based and newly synthesized alternative OER catalysts are produced in powder form, necessitating their dispersion as an ink formulation and subsequent immobilization on a PEM and hot pressing on a porous transport layer (PTL) to achieve catalyst-coated membranes (CCMs).^10^ The testing of these catalyst materials in real devices (membrane electrode assemblies, MEAs) is desirable but not routinely applied due to its prohibitively expensive and time-consuming nature.^9^ At lab-scale, such catalyst assemblies are most frequently studied through drop-casted nanoparticle-based thin-film rotating disk electrode (NP-TF-RDE) aqueous model systems (AMSs) that are straightforward and less time and material demanding.^10−12^ The value of the NP-TF-RDE-extracted results depends on their transferability to technical MEA approaches that closely resemble the actual application.^11,13^ However, a current challenge that puzzles researchers is the discrepancy of catalyst stability metrics derived from MEA and AMS studies.^7,11,13−19^ Typically, the materials exhibit three to four orders of magnitude longer lifetimes when tested in MEAs than when conducted through AMSs.^11,13,17^ This undermines the efforts aimed at bridging AMS-derived stability assessments with valid projections to practical platforms. Rigorous and insightful work has been devoted to the elucidation of this issue, and debate about the reasons behind the disagreement dominates current research in the OER community.

Spöri et al.^20^ have drawn attention to the importance of distinguishing between apparent and actual electrocatalyst material degradation in AMS studies. This categorization differentiates between intrinsic and operational material stability, the latter being assigned to changes taking place in situ on the solid–liquid interface due to blockage of active sites, catalyst detachment, and substrate oxidation/passivation. Currently, there is consensus among researchers that (i) substrate degradation (backing electrode material in RDE experiments) due to highly oxidizing OER conditions and (ii) blockage of catalyst active sites by electrogenerated O_2_ bubbles are the two main reasons for the apparent catalyst degradation in OER studies that lead to the overestimation of material instability in RDE-based AMS.^15,21,22^ On the one hand, the substrate degradation challenge has been tackled by Cherevko et al.^21^ and Seitz et al.,^23^ who combined electrochemical with direct surface characterization studies to demonstrate that commonly used RDE substrate materials that act as catalyst backing supports fail to fulfill inertness and oxidize under water oxidation conditions. The catalyst eventually becomes electrically isolated following the increase in the surface contact resistance, thus shortening the stability of the electrode assembly. On the other hand, El-Sayed et al.^11,15,22,24,25^ have provided insightful contributions to the bubble-related issue and demonstrated through comprehensive electrochemical investigations that the classical OER screening strategies based on NP-TF-RDE studies suffer from the accumulation and inefficient detachment of microscopic O_2_ bubbles inside the porous structure of powder-based catalyst materials. The authors have suggested that the decline in catalyst performance and overestimation of material instability in these systems originate mainly from the consequent decrease in material utilization (shielding of active sites by O_2_ bubbles).^24^ Thus, they proposed that prevention of the bubble accumulation within the catalyst layer in NP-TF-RDE studies would enable the design of proper stability protocols for reliable OER catalyst benchmarking.^25^ Despite the independently developed understanding of these two processes (e.g., substrate passivation and catalyst blockage by O_2_ bubbles), their interplay and relative impact on the intrinsic and operational material stability have not yet been established, and elucidation of whether they occur simultaneously or sequentially in the course of NP-TF-RDE-based OER investigations remains an open question. This ambiguity, along with the stability discrepancy between AMS and MEA results, has stimulated criticism of the ubiquitous RDE-based screening method and its suitability for OER catalyst durability benchmarking. While some reports discourage its utilization for such determinations,^15,25^ others continue endorsing it, provided that its limitations are accounted for.^11,14,26^

In this contribution, we employ an inverted RDE (iRDE)-based approach coupled with online gas quantification that excludes the influence of bubble retention on the surface of the catalyst and allows the dissection of the electrode assembly degradation process occurring during NP-TF-RDE-based OER stability studies. This enables unambiguous identification of the origins of the apparent catalyst degradation and allows accurate quantification of the intrinsic catalyst material loss. In addition, we propose experimental measures to mitigate or circumvent NP-TF-RDE-related limitations when the technique is to be applied to OER stability assessment. We suggest that these findings advance the transferability between the AMS and MEA approaches.

## Methods

### Preparation of NP-TF-(i)RDE Electrodes

Two inks were prepared by dispersing a commercially available IrO_*x*_ catalyst (Alfa Aesar, iridium(IV) oxide, Premion, 99.99% metals basis, Ir 84.5% min, MFCD00011065) in a 3:1 (v/v) ultrapure H_2_O:isopropanol (Millipore, 18.2 MΩ cm, 3 ppb TOC and HPLC grade, VWR Chemicals) mixture. The dispersion was sonicated at room temperature for 5 min. 10 wt % (based on the amount of Ir) of Nafion dispersion (D1021, Fuel Cell Store) was added to the mixture, and the resulting ink was sonicated for 5–10 more minutes. The corresponding concentrations were 0.196 and 1.963 mg_Ir_ mL^–1^. These two concentrations were chosen to drop-cast a sufficient volume of ink covering the entire electrode surface. Glassy carbon (GC), Au, and Ti disk inserts (4 mm in height and 5 mm in diameter, Pine Research) were pressed into laboratory-made poly(tetrafluoroethylene) (PTFE) or polysulfone (PSU) shrouds, mirror-polished with 0.3 μm alumina suspension (MasterPrep, Buehler), thoroughly rinsed with ultrapure water, sonicated for 20 min in isopropanol, immersed in boiling ultrapure water for 20 min, rinsed, and dried with Ar (99.999%, Carbagas, Switzerland). The Au electrodes were additionally electrochemically polished by oxidation at 2 V in 0.1 M H_2_SO_4_ for 20 s, followed by immersion in 0.1 M HCl for 5 min and thorough rinsing with ultrapure water. 10 and 5 μL of the beforehand sonicated 0.196 and 1.963 mg_Ir_ mL^–1^ inks were drop-casted on the surface of the electrodes to achieve 10 and 50 μg_Ir_ cm^–2^ catalyst loadings, respectively. The electrodes were dried overnight under an air atmosphere at room temperature. The quality of the electrode was assessed with a light microscope (VHX-6000, Keyence).

### Preparation of Electrochemically Deposited IrO_*x*_ (ECD_IrOx_) Layers on Ti-(i)RDE Electrodes

To demonstrate the improved catalyst stability assessment based on compact thin IrO_*x*_ layers electrodeposited on Ti-RDE substrates, we employed the synthesis proposed by Choe et al.^27^ Briefly, the deposition bath was composed of 10 mM IrCl_3_ hydrate (IrCl_3_·*x*H_2_O, 99.8% metals basis, Alfa Aesar), 100 mM hydrogen peroxide (H_2_O_2_, 31% BASF), 40 mM oxalic acid ((COOH)_2_·2H_2_O, ACS reagent, Sigma-Aldrich), and 340 mM potassium carbonate (K_2_CO_3_, ReagentPlus, Sigma-Aldrich). This solution was stirred for 3 days prior to deposition to allow stabilization and had a pH value of 10.3. The Ti-RDE tips were mirror-polished using diamond suspension (0.3 μm, Bühler), thoroughly rinsed, sequentially sonicated in ultrapure water and isopropanol, rinsed, and dried in air. The deposition was carried out using the experimental setup shown in Figure S1 to avoid the potential buildup of O_2_ bubbles on the sample surface that would induce porosity in the IrO_*x*_ layer. A saturated calomel reference electrode (SCE) and a Ti foil counter electrode were used to conduct potentiostatic electrodeposition at 0.7 V vs SCE for 5 min. The samples were then thoroughly rinsed and stored for galvanostatic OER screening and scanning electron microscopy-energy-dispersive X-ray spectroscopy (SEM-EDS) characterizations. Information on the catalyst loading and extracted stability metrics from galvanostatic OER screening is provided in Table S1.

### Electrochemical RDE and iRDE Measurements

The RDE electrochemical experiments were carried out using a conventional three-electrode glass cell. The iRDE instrument and the corresponding H-type glass cell are described in ref (28) (see also Supporting Information Note 1). The NP_IrOx_-TF-(i)RDE and ECD_IrOx_-TF-(i)RDE anodes were used as working electrodes during the galvanostatic OER electrolyses. “Leakless” Ag/AgCl_3M_ electrodes and GC rods were used as reference and counter electrodes, respectively. The reference electrodes were thoroughly rinsed with ultrapure water before being inserted into the electrolyte. The counter electrodes were sequentially wiped with precision wipes (Kimtech Science) and isopropanol, thoroughly rinsed with ultrapure water, immersed in boiling water for 30 min, and rinsed before insertion into the catholyte compartment. The two cell compartments were separated by means of glass frits or Nafion ion-exchange membranes (Nafion 117, Sigma-Aldrich). The 0.1 M H_2_SO_4_ electrolytes (96%, Suprapur, Merck) were saturated with O_2_ (99.9995%, Carbagas, Switzerland) for at least 30 min before conducting electrolyses. ECi-210 potentiostats controlled by EC4 DAQ 4.2 software (Nordic Electrochemistry ApS) were used for all electrochemical measurements. The *iR* drop was measured throughout the measurements and all potential transients shown were corrected postelectrolysis. No *iR* drop compensation was applied for the potentiostatic oxygen reduction reaction (ORR) cycles. Note that the limiting currents reached in these experiments are below the 100 μA threshold, and the corresponding *iR* drop was therefore very low. The potentials were converted to the RHE scale by using eq 1:
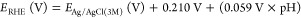
1Apart from selected cases, all galvanostatic OER electrolysis performed with (i)RDE anodes were carried out at *f* = 1000 rpm. Selected experiments were coupled to a gas chromatograph, in which case Ar was used as the carrier gas.

### Electrochemical OER Measurements on Stationary Half-Covered GC Electrodes

A series of stationary GC electrodes (0.8 × 1.25 cm^2^) were prepared by drop-casting IrO_*x*_ catalyst on their upper half surfaces (50 μg_Ir_ cm^–2^). Both catalyst-functionalized and catalyst-free surfaces were immersed in the O_2_-saturated 0.1 M H_2_SO_4_ electrolyte and subsequently subjected to galvanostatic OER electrolysis at 10 mA cm^–2^ (relative to the whole immersed surface) for distinct time periods while recording the bubble evolution with a camera positioned perpendicularly to them. Selected experiments were coupled to a gas chromatograph, in which case Ar was used as carrier gas.

### Inductively Coupled Plasma Mass Spectrometry (ICP-MS)

The amounts of dissolved Ir and Au induced by galvanostatic OER electrolysis were determined by ICP-MS (PerkinElmer, NexION 2000). Aliquots were taken prior to electrolysis and after selected times from the H-type cells once the galvanostatic OER had been started. 500 μL aliquots were digested in a mixture of 300 μL of concentrated HNO_3_ (69.3%, <1 ppb EMCE, BASF) and 173 μL of concentrated HCl (36%, <1 ppb EMCE, BASF) at 65 °C for 3 h. Ultrapure water was added to achieve a final solution of 5.0% HNO_3_ (v/v). Quantitative standards containing 10, 100, 1000, and 10,000 ppt of the corresponding metals were prepared to build a calibration curve (Au ICP standard, 1000 mg L^–1^ in 10% HCl, PerkinElmer; Ir ICP standard, 1000 mg L^–1^ in 7% HCl, Merck; Pt ICP standard, 1000 mg L^–1^ in 5% HCl, Sigma-Aldrich; Ti ICP standard, 1000 mg L^–1^ in H_2_O, Merck). Twenty ppb Co and Re were used as internal standards during the ICP-MS analysis.

### Calculation of *S*-Numbers (*S*_ICPMS_), Pseudo-S-Numbers (*S*_gal_), and Catalyst Lifetimes from Galvanostatic OER Screenings

For the calculation of *S*-numbers, the amount of electrogenerated oxygen was derived from cumulative Faradaic charge between the start of the measurements and the time at which the potential plateaus commenced. For this calculation, we assumed that no Faradaic process other than the OER was of relevance during this time. Formal ICP-MS-derived *S*-numbers (*S*_ICPMS_) were calculated by dividing the moles of oxygen produced by the moles of dissolved Ir determined by ICP-MS.

In addition, pseudo-*S*-numbers (*S*_gal_) were calculated from the electrochemical data, assuming that all of the catalyst loaded on the employed RDE tips was consumed at the moment the potentials attained the substrate-dependent plateaus.

Lifetimes of the catalyst were calculated according to eq 2:^7^
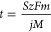
2where *t* is the lifetime of the catalyst, *S* is the stability number, *z* is the number of electrons per evolved molecule of oxygen, *F* is the Faraday constant (96,485 C mol^–1^), *m* is the loaded mass of iridium (g cm^–2^), *j* is the applied current density (A cm^–2^), and *M* is the molar mass of iridium (192.2 g mol^–1^). All results are displayed in Table S1.

### Identical Location (IL) SEM and EDS Characterization

Morphological and elemental characterization of the prepared IrO_x_-coated (i)RDE tips was carried out by SEM imaging and EDS experiments. IL imaging was performed before (for the as-prepared electrodes) and after sustained defined OER time intervals at selected applied current densities. For postelectrolysis SEM-EDS characterization, the anodes were immersed for 10 min in ultrapure water and sequentially rinsed and dried under ambient conditions. The analysis was conducted with a Zeiss Gemini 450 scanning electron microscope with both InLens secondary electron and backscattered electron detectors (InLens SE and BSD detectors, respectively). An accelerating voltage of 5 kV and a current of 70–200 pA were applied at a working distance of 5–7 mm. The BSD detector enabled clear identification of the IrO_x_ catalyst on the surface of the GC, Au, and Ti substrates due to the high sensitivity to the atomic number of the elements being imaged. However, the images acquired with the InLens SE detector provided better morphological resolution of the catalyst. The use of both imaging operational modes coupled with EDS analysis made it possible to track morphological catalyst changes induced by OER electrolysis. AZtec 6.1 software (Oxford Instruments) was used to acquire the EDS spectra and surface elemental mappings. An acceleration voltage of 10 kV and a current of 1.2 nA were applied at a working distance of 8.5 mm.

### Raman Spectroscopy

Raman spectra were collected in a backscattering geometry using a LabRAM HR800 confocal microscope (Horiba Jobin Yvon). Prior to the measurements, the instrument was calibrated using a silicon wafer standard at a 520.6 cm^–1^ Raman shift. The measurements were conducted with an 8 mm working distance between the objective lens (Olympus LMPLFLN, 50× magnification) and the sample, utilizing a numerical aperture of 0.1 to focus a He–Ne laser beam (excitation wavelength of 633 nm, power of 3 mW) on the sample. Spectra were obtained from at least four distinct locations on both catalyst-free and catalyst-rich sample areas before and after the OER electrolysis. The data corresponding to each applied condition was normalized to the highest signal intensity within the relevant Raman shift range and averaged. The corresponding error bars demonstrate the minimal deviation among the measurements for each sample under specific electrolysis conditions.

### Gas Chromatography Measurements

The electrogenerated gaseous products were analyzed online by a gas chromatograph (GC 8610C, SRI Instruments) equipped with packed HayeSep D and packed Molsieve 5A columns. A thermal conductivity detector (TCD) and flame ionization detector (FID) were applied for the quantification of the formed O_2_ and CO and CO_2_, respectively. The gaseous products were conducted from the headspace of the electrolysis cell to the gas sampling loop (100 μL) of the gas chromatograph using Ar (99.999%, Carbagas, Switzerland) as a carrier gas in sequential intervals of 3 min (O_2_) or 5–10 min (CO and CO_2_) during galvanostatic OER measurements. Independent experiments were carried out for O_2_ and CO and CO_2_ analysis. Calibration gas mixtures (Carbagas, Switzerland) with concentrations ranging from 100 to 3000 ppm were used for gas quantification. The continuous flow of the carrier gas through the electrolysis cell carried volatile reaction products from the headspace into the sampling loops of the gas chromatograph. The Faradaic efficiency of oxygen was determined, as explained in ref (29).

## Results

### Inverted RDE for Exclusion of Catalyst Surface Blockage by Electrogenerated O_2_ Bubbles

**Figure 1:**
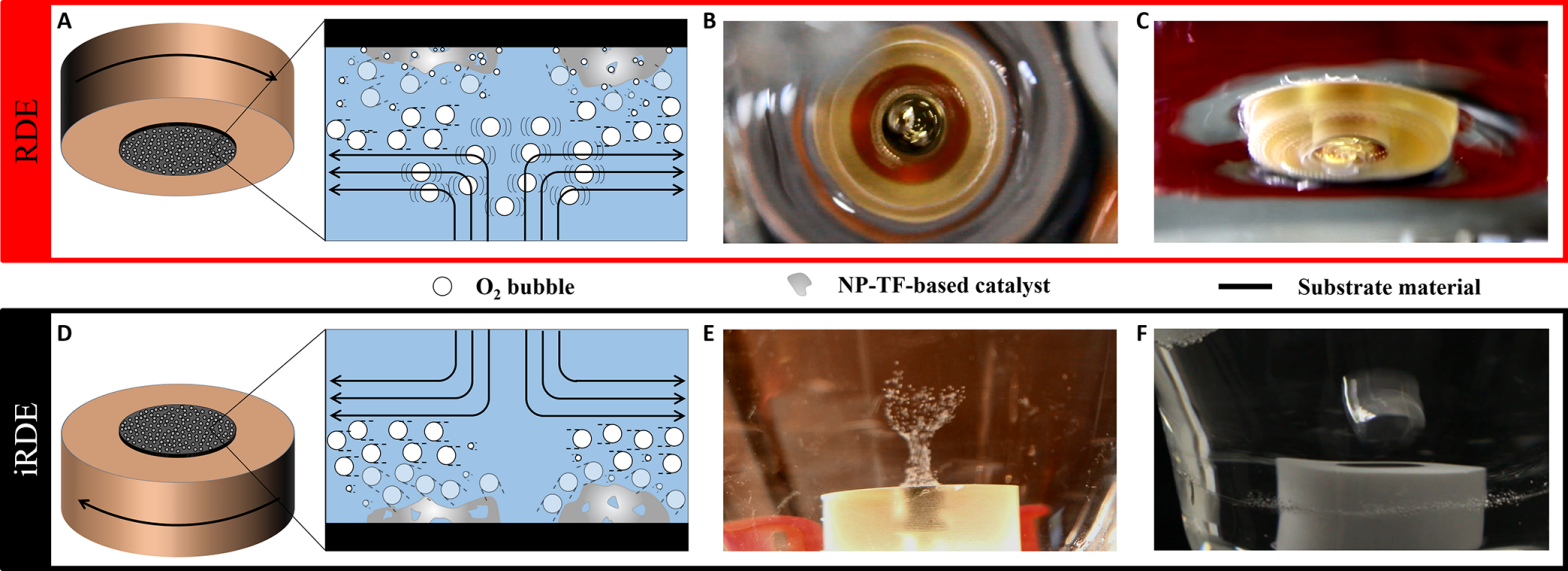
(A) Schematics of bubble formation and incomplete detachment and removal from the electrocatalyst surface typically encountered when conducting NP-based OER experiments with an RDE instrument. (B, C) Pictures of catalyst-coated RDE tips taken in the course of galvanostatic OER screenings. (D) Schematics of bubble formation and efficient detachment and removal from the electrocatalyst surface when conducting NP-based OER experiments with an iRDE instrument. (E, F) Pictures of catalyst-coated iRDE tips taken in the course of galvanostatic OER screenings. The pictures in (B), (C), and (F) were taken for electrodes rotating at 1000 rpm, while the one in (E) was taken without applying forced convection. All pictures are from electrolysis experiments with NP-based IrO_x_ coated electrodes (loading: 10 μg_Ir_ cm^–2^) at a current density of 30 mA cm^–2^ in a 0.1 M H_2_SO_4_ solution.

To shed light on the issues of NP-TF-RDE-based catalyst stability tests, we employed the stability approach proposed by the Joint Center for Artificial Photosynthesis (JCAP).^6^ The protocol regards electric potential as a stability metric and has intensively been employed for over a decade due to its straightforwardness and low catalyst loading required. The protocol relies on monitoring the potential developed at the catalyst–electrolyte interface while carrying out OER electrolysis at a constant anodic current density. The approach ascribes monotonous potential increments occurring in the course of the OER to gradual catalyst degradation and subsequent exponential increases to complete catalyst depletion.^30,31^ Following depletion, the potential plateaus at a value that depends on the substrate material employed. El-Sayed et al. advocate that in a conventional RDE configuration, this electrochemistry-derived stability metric is not reliable because oxygen microbubbles unavoidably accumulate within the catalyst layer and gradually block the surface of the catalyst material.^15,22,24^ In this scenario, the complete blocking of the catalyst surface is manifested in the galvanostatic OER by the achievement of a cutoff potential (when the above-mentioned abrupt potential increase sets in) that is mistakenly assigned to the catalyst’s end-of-life. Figure 1A illustrates the impaired dynamics of bubble detachment operating in conventional RDE setups that lead to catalyst shielding (see Supporting Information Note 2). The scheme shows that although most macroscopic bubbles detach from the catalyst surface, a fraction of them remains in front of the electrode–electrolyte interface, disrupting the free path of incoming electrolyte and outgoing O_2_, causing unfavorable mass transport as well as ohmic and kinetic effects.^32,33^ More importantly, microbubbles generated inside the porous structure of the catalyst material are very likely not to detach completely. This leads to the transient blocking of active sites and induces higher local current densities on the bubble-free surface when a galvanostatic OER is applied. Figure 1B,C shows representative photographs of downward-facing NP-TF-RDE electrodes taken during galvanostatic OER electrolysis at high current densities. Clearly, a bubble cloud caused by deficient gas removal occupies a substantial fraction of the interface near the anode surface.

To circumvent these bubble-related issues, we resorted to an experimental approach, namely, the iRDE, that overcomes the inherent limitations of the standard RDE for gas-evolving processes.^34^Figure 1D schematically shows that unlike the RDE, the iRDE configuration exploits the buoyancy exerted on the generated O_2_, achieving its efficient detachment from the catalyst surface and preventing, in addition, its buildup in front of the polarized anode.^28,35,36^Figure 1E exemplarily shows that even in the absence of forced convection, the produced oxygen readily detaches from the catalyst-coated iRDE surface (Supporting Information Video S1). The bubble management achieved with the iRDE is so effective that it also induces the removal of bubbles occasionally adhering to the hydrophobic electrode shroud (Figure 1F and Supporting Information Video S2). Importantly, besides being amenable to the same mathematical treatment as standard RDEs, the instrument operates airtight when coupled to online gas chromatography.

**Figure 2:**
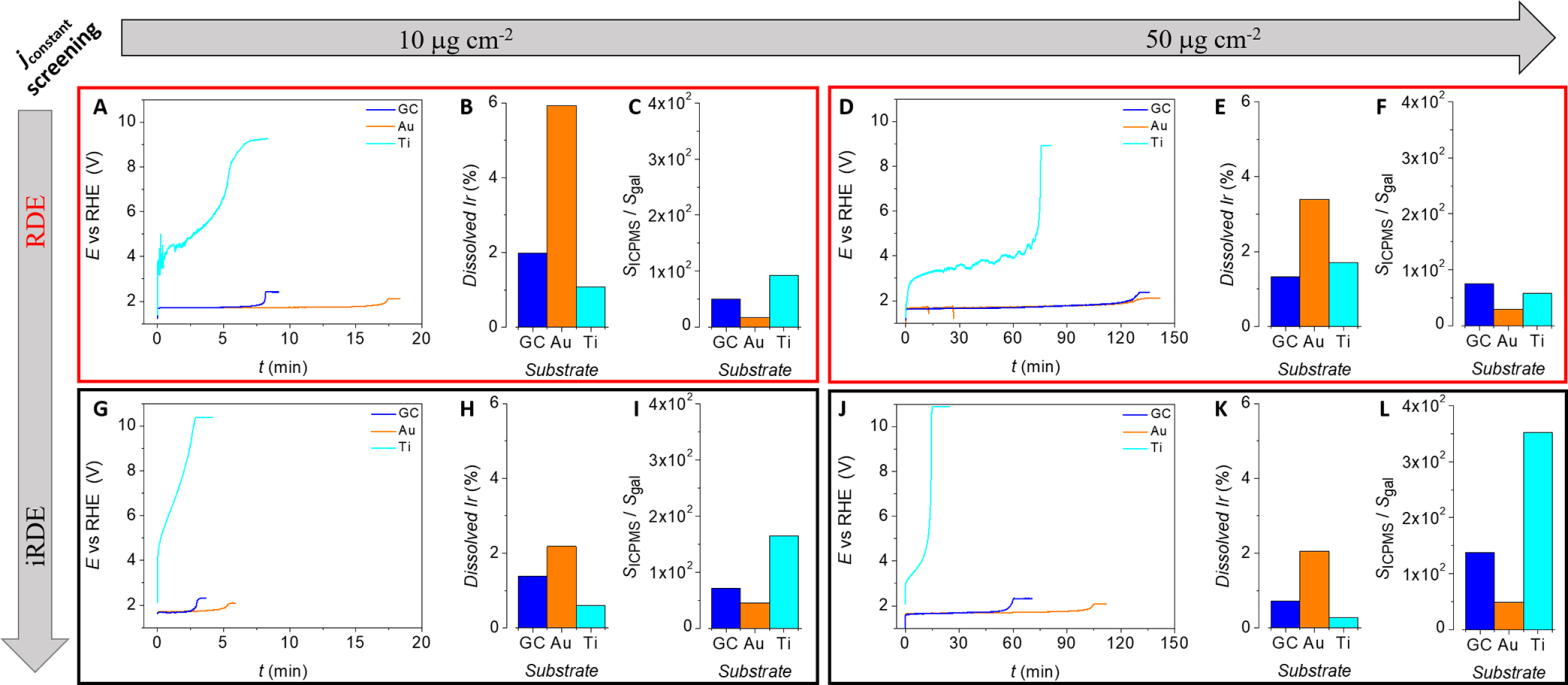
(A–C) and (D–F) Potential transients, dissolved amounts of Ir and *S*_ICPMS_ to *S*_gal_ ratios of galvanostatic RDE experiments with 10 and 50 μg_Ir_ cm^–2^ catalyst loading, respectively. (G–I) and (J–L) Potential transients, dissolved amounts of Ir and *S*_ICPMS_ to *S*_gal_ ratios of galvanostatic iRDE experiments with 10 and 50 μg_Ir_ cm^–2^ catalyst loading, respectively. All experiments were performed at a specific current density *j* = 30 mA cm^–2^ and a rotational frequency *f* = 1000 rpm in an O_2_-saturated 0.1 M H_2_SO_4_ supporting electrolyte. Pseudo-S-numbers (*S*_gal_) were calculated from the electrochemical data, assuming that all of the catalysts loaded on the employed (i)RDE tips were consumed at the moment the potentials attained the substrate-dependent plateaus.

As this approach excludes the massive blockage of the catalyst material by bubbles, we should, according to the findings of El-Sayed et al., be able to determine the intrinsic catalyst stability by the galvanostatic stability protocol.^24,25^ For the sake of comparison, we first conducted reference RDE experiments that were contrasted with the iRDE-derived results. Figure 2A displays potential transients from RDE baseline experiments with a state-of-the-art IrO_x_ catalyst at a specific current density *j* = 30 mA cm^–2^ (catalyst loading 10 μg_Ir_ cm^–2^, mass-normalized current 3 A mg_Ir_^–1^). Three substrate materials were used as backing supports, namely, GC, Au, and Ti. This selection obeys the fact that GC is the most frequently used substrate material in such durability tests, Au has proven beneficial to extend the lifetime of the catalyst, and Ti is the actual material that is brought into contact with the catalyst in technical approaches (MEAs and PEMWEs).^37^Figure 2A demonstrates a remarkable effect of the substrate on the electric potentials developed at the catalyst–electrolyte interface and the times elapsed before the attainment of the sharp potential jump. This indicates that the physicochemical properties of the substrate influence the dynamics of electrode degradation. The initial potential (*E*_initial_, observed immediately after having surpassed the surface charging) and plateau values (*E*_plateau_, attained after the potential jump reaches its maximum) are provided in Table S1. The times at which the plateaus were reached followed the sequence *t*_Ti_ < *t*_GC_ < *t*_Au_ and amounted to only a few minutes, in agreement with studies carried out under similar AMS conditions.^15^ In contrast, catalyst durabilities extracted from MEA investigations typically reach orders of magnitude longer lifetimes.^13^ Motivated by this stability inconsistency between NP-TF-RDE- and MEA-based results, alternative stability benchmarking methods relying not only on electrochemical data have been introduced that identify noble-metal dissolution as the primary instability mechanism.^38−40^ The stability (*S*)-number and activity-stability factor (ASF) proposed by Geiger et al.^7^ and Kim et al.^8^ were devised as intrinsic material stability metrics for noble metal-based electrocatalysts and report on noble-metal dissolution normalized to OER performance. Both metrics are very similar and have attracted increasing interest and have been applied to characterize the stability of a growing number of materials. In the following, we employ the *S*-number metric as it is more suitable to evaluate intrinsic catalyst stability from galvanostatic OER screening and allows estimation of the electrocatalyst lifetime. The dissolved amounts of Ir were obtained through post-mortem ICP-MS analysis of the electrolyte solutions used for all presented galvanostatic OER experiments in Figure 2. Interestingly, relative to the initial catalyst loading on the samples, very low fractions of dissolved Ir were found (Figure 2B and Table S1). The amount of electrogenerated oxygen was derived from cumulative Faradaic charge between the start of the measurements and the time at which the potential plateaus commence. For this calculation, we assume that no Faradaic process other than the corresponding OER is of relevance during this time. ICP-MS-derived *S*-numbers were determined according to the relation *S*_ICPMS_ = *n*_O_2__/*n*_Ir_ and are presented in Table S1. Additionally, pseudo-*S*-numbers (*S*_gal_) were calculated from the electrochemical data, assuming that all of the catalyst loaded onto the employed RDE tips was consumed at the moment the potentials attained the substrate-dependent plateaus (Figure S3). Figure 2C displays *S*_ICPMS_ to *S*_gal_ ratios that exemplarily illustrate the underestimation of catalyst stability determined by electrochemical means alone. Next, we performed complementary experiments with a 5-fold increase in catalyst concentration (50 μg_Ir_ cm^–2^, applied mass-normalized current 0.6 A mg_Ir_^–1^). Although higher material loadings led to higher *S*_ICPMS_ and *S*_gal_, the obtained stability figures still failed to meet what is typically observed in MEA studies (Figure 2D–F and Table S1). Note that the 5-fold increase in catalyst loading did not induce 5-fold increased stability figures. Indeed, recent investigations by Knöppel et al.,^13^ based on aqueous scanning flow cell (SFC)-ICP-MS measurements found no direct correlation between metal catalyst loading and its dissolution rate.

As El-Sayed et al. suggest, the prevention of microbubble retention within the catalyst layer in NP-TF-RDE measurements should enable accurate prediction of catalyst lifetimes in OER investigations that should match MEA results.^25^ Therefore, experiments under the same conditions as those applied for the results shown in Figure 2A–F but employing the iRDE were carried out (Figure 2G–L and Table S1). In contrast to the expected increased stability metrics that should reflect the true catalyst stability due to the prevention of microbubble retention within the catalyst layer, the iRDE-derived chronopotentiometric results show that despite exhibiting lower *E*_initial_ (Table S1), the overall electrochemical performance is rather close to that obtained with the conventional RDE approach. Indeed, in all cases, the cutoff potential was reached at slightly shorter electrolysis times with the iRDE than with the RDE. The reason for this apparently counterintuitive finding will be explained in the final section of this article. However, the corresponding amounts of dissolved Ir were lower when employing the iRDE, and the *S*_ICPMS_ to *S*_gal_ ratios showcased a larger disagreement between the electrochemically obtained stability number and the formal *S*_ICPMS_. These findings indicate that the overall shape of potential transients and the times at which the cutoff potential is reached in NP-TF-RDE-extracted OER experiments are not rooted in the catalyst surface shielding by the O_2_ bubbles. Therefore, bubble accumulation on the catalyst surface does not represent the main reason for the disparities between conventional AMS and MEA studies.

**Figure 3:**
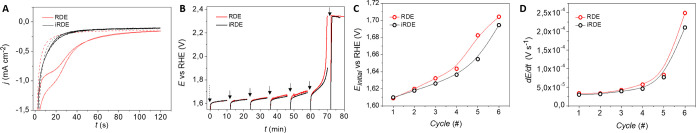
(A) and (B) Selected 2 min ORR steps at 0.2 V vs RHE followed by alternating 10 min OER steps at 10 mA cm^–2^ in an NP-TF-RDE (red) and NP-TF-iRDE (black) setups. The dotted curves in (A) correspond to the initial ORR steps before any OER step was measured. The solid lines are selected chronoamperograms measured after galvanostatic OER cycles were applied but before the appearance of the drastic potential jump. The black arrows in (B) indicate the times at which the ORR cycles were applied either before (dotted) or between (solid) galvanostatic OER steps. (C) and (D) Initial potential (*E*_initial_) and potential slope (d*E*/d*t*) transients extracted from the individual OER cycles shown in (B). To avoid the influence of surface charging, (*E*_initial_) and (d*E*/d*t*) were taken, excluding the first 2 min of the corresponding OER cycle. The IrO_x_ loading was 50 μg_Ir_ cm^–2^ on the GC-(i)RDEs, and the electrolysis was carried out at *f* = 150 rpm in an O_2_-saturated 0.1 M H_2_SO_4_ supporting electrolyte.

To further validate this hypothesis, dedicated experiments were conducted, in which catalyst-coated GC-RDE and GC-iRDE anodes were subjected to alternating 2 min long potentiostatic oxygen reduction reaction (ORR) and 10 min long galvanostatic OER cycles (0.2 V vs RHE and 10 mA cm^–2^, respectively) that were sustained until the cutoff potential and following plateau value were reached. This way, if O_2_ bubbles were still to be retained within the catalyst pores in the course of the OER, each subsequent ORR cycle would reduce them completely and the continuation of the galvanostatic OER would resume with a bubble-free catalyst layer.^41^Figure 3A shows ORR potentiostatic cycles for RDE (red curves) and iRDE (black curves). The first ORR cycles were recorded before any galvanostatic OER event took place (dotted lines). These current density transients show a large cathodic current within the first few seconds that we attribute to double-layer charging and a possible weak contribution from the reduction of trapped O_2_ bubbles within the catalyst left inside by the drop-cast preparation method.^41^ After about 30 s, the current falls to a mass transport-limited value, indicating that by the end of the ORR cycle, the catalyst layer is oxygen-free (the experiments were carried out with an O_2_-saturated electrolyte). Note that regardless of the specific RDE version used (i.e., RDE or iRDE), the first ORR cycles almost overlap. The galvanostatic OER step series were then started (Figure 3B). Selected ORR cycles recorded after the galvanostatic OER series had been started but prior to the emergence of the exponential potential increase are also shown in Figure 3A (solid lines). Contrary to the ORR current transients obtained with the iRDE that overlap with the one recorded before the start of the OER cycles, the RDE-derived current densities exhibit much larger currents that slowly decay before the attainment of the mass transport-limited value. This proves that when using the RDE, a fraction of the generated bubbles during an OER step unavoidably remains confined inside the pores of the catalyst layer and is reduced in the following ORR cycle without mass-transport limitations, thus giving rise to larger currents than with the iRDE. This demonstrates that the iRDE approach completely removes the electrogenerated O_2_ bubbles from the catalyst material formed during galvanostatic OER stability tests. Conversely, conventional RDE experiments are inevitably undermined by the above-mentioned limitations. The galvanostatic steps shown in Figure 3B also demonstrate that if there is efficient removal of bubbles (e.g., by alternating ORR–OER cycling), (i) the RDE performance approaches rather closely that of the iRDE and (ii) the RDE can be used to obtain quasi bubble-free electrochemical signals. We acknowledge a minor effect of trapped bubbles inside a porous electrocatalyst on the galvanostatic signals recorded with an RDE during the OER experiments. Figure 3C,D shows the time evolution of the *E*_initial_ and potential slope (d*E*/d*t*) extracted from the individual OER cycles shown in Figure 3B. Clearly, slightly higher potentials must be developed at the catalyst–electrolyte interface when conducting an RDE rather than an iRDE experiment to maintain a constant current flow. Therefore, the RDE compromises the true stability of the catalyst more severely than the iRDE when conducting the galvanostatic OER (ICP-MS data in Figure 2). The trends shown in Figure 3C,D indicate that even at the very beginning of every OER step, a certain amount of the generated O_2_ is retained inside the catalyst material supported on an RDE. Nevertheless, the results addressed in this section conclusively demonstrate that the assignment of stability disagreement between purely electrochemically obtained NP-TF-RDE and MEA data does not mainly originate from the accumulation of bubbles within the catalyst layer in the course of water oxidation.

### Substrate Passivation: The Main Reason for Different Catalyst Durability Prognoses Given by Electrochemical and ICP-MS Approaches

To clarify the ambiguity of the purely electrochemically derived results that misalign with the ICP-MS-derived data, we provide in Figure S4 a complementary physicochemical characterization of anode specimens analyzed prior to and after the applied OER electrolyses. We employed the so-called identical location (IL) SEM- and EDS-based techniques^42−44^ and Raman spectroscopy. These analyses provided the structural and compositional evolution of the electrocatalyst by comparing its morphology and composition at the same sample location before and after being subjected to electrolysis that lasted a few minutes beyond the appearance of the potential plateau. Figure S4A–F displays representative IL-SEM analysis corresponding to NP-TF-iRDE samples with 50 μg_Ir_ cm^–2^ catalyst loading and whose electrochemical screening is shown in Figure 2J. The high magnification SEM micrographs in Figure S4A–C show that regardless of the substrate material, the nanostructure of the catalyst undergoes minor changes as a result of the electrolysis. Besides, contrary to what the potential transients suggest, significant amounts of the IrO_x_ catalyst remained on all three sample surfaces despite having sustained the electrolysis of the OER beyond the appearance of the abrupt potential jump. On the other hand, the catalyst loss observed on the sample surfaces also mismatches the extremely low material dissolution determined by ICP-MS measurements (Figures 2K,L, S4D–F and Table S1). Selected catalyst and substrate morphological features that are recognizable both before and after the electrolysis are enclosed by continuous and discontinuous yellow shapes in Figure S4A–F, respectively. Concerted consideration of ICP-MS and IL-SEM-EDS results indicates that the catalyst undergoes minimal electrochemical dissolution during the electrolysis of the OER at the applied mass current density of 0.6 A mg_Ir_^–1^ but is affected by partial detachment from the underlying substrate to extents that depend on the backing electrode material supporting them. Clearly, the detachment rate is more substantial for the catalyst supported on Au than on Ti and GC. Interestingly, as demonstrated in Figure S5, the material detachment is clearly milder for samples with 10 μg_Ir_ cm^–2^ catalyst loading (gold included) subjected to higher mass current densities of 3 A mg_Ir_^–1^. Thus, the loss of material through mechanical detachment might be related to weak interparticle binding undermined by vigorous O_2_ evolution or applied forced convection rather than poor adhesion between the catalyst and the supporting substrates.

Several groups have reported on the operational degradation of the substrate materials typically used for OER screening in acidic media, which do not provide sufficient chemical inertness.^9,21,23^ GC with a thermodynamic oxidation potential of 207 mV vs RHE is known to suffer from oxidation/passivation at the relevant potentials applied in electrochemical water oxidation.^45^ Analogously, Au undergoes surface oxidation and anodic dissolution at ca. 1250 mV vs RHE.^9,46−48^ Ti, the material that supports the catalyst on the anode side of PEMWEs, is known to easily oxidize and form a passive surface oxide layer at ca. 1000 mV vs RHE.^21,27^ The IL-SEM-EDS analysis on the GC-supported catalyst in Figure S4D demonstrates that the substrate surface experiences structural degradation induced by the galvanostatic electrolysis. The red arrows point to crevices along the postelectrolysis sample surface that can reach above 10 μm in length. Similar characteristics are observed for the GC-RDE and GC-iRDE samples that were subjected to the more stressing ORR–OER cycling (Figures 3 and S6).^49^ Complementary Raman spectroscopy was performed before and after electrolysis on catalyst-free and catalyst-rich positions to confirm the presence of the catalyst and reveal the nature of the substrate surface degradation. Figure S4G displays Raman spectra of the as-prepared and galvanostatically screened (green and blue spectra, respectively) anodes. The peak signatures at 550 and 720 cm^–1^ corresponding to IrO_x_ confirm that the electrocatalyst is abundantly present on the GC surface after the OER tests and support the IL-SEM-EDS results. The Raman signals in the range of 1200–1650 cm^–1^ are GC-related. The observed relative increase of the graphite (G) to defect (D) bands (at 1580 and 1360 cm^–1^, respectively) induced by the OER is attributed to increased amorphization due to molecular structures that are formed by oxidation processes.^50−53^ The increase in signal intensity and broadening at the local minimum between the D and G bands is also indicative of amorphous molecular structures, fragments, or functional groups that form upon oxygen uptake during the oxidation process.^9,23,54,55^ Apart from the partial detachment of the catalyst material, our IL-SEM-EDS investigations conducted on Au- and Ti-supported anodes do not indicate morphological transformations of the substrates (Figure S4E,F). Nonetheless, the Raman characterizations show that these two substrate materials also oxidize following the conducted OER electrolysis. The spectrum of the postelectrolysis Au-supported anode (orange curve, Figure S4H) exhibits a convoluted signal composed of superimposed IrO_x_ and the broad AuO_x_ (593 cm^–1^) fingerprints.^56^ The stressed Ti-backed electrode displays characteristic Raman signatures of IrO_x_ and anatase TiO_2_ (146 and 634 cm^–1^, Figure S4I).^57,58^ These ex-situ surface characterizations coupled with the ICP-MS findings and the exclusion of bubble accumulation inside the catalyst layer addressed in the previous section demonstrate that the NP-TF-RDE-based galvanostatic stability test fails to determine the true electrocatalyst degradation brought about by OER screenings and reflects instead the failure of anode assemblies due to the employment of catalyst-coated corrosion-prone substrate materials.^9,23,54,55^ Note that the key finding in the study of El-Sayed et al.^24^ was obtained on a corrosion-proof polycrystalline Ir-RDE electrode that concealed the issues addressed in this section and was only influenced by intrinsic limitations of the conventional RDE approach in gas-evolving processes (Supporting Information Note 2).

### Substrate Degradation Mechanism in the RDE-Based Galvanostatic OER

**Figure 4:**
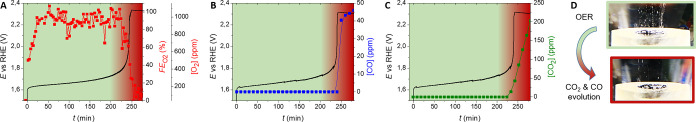
(A)–(C) Online detection of O_2_, CO, and CO_2_ by gas chromatography during OER screening on IrO_x_-coated GC-iRDEs. (D) Pictures of an IrO_x_-coated GC-iRDE taken before and after attainment of the cutoff potential during OER electrolysis (green and red frames, respectively). Clearly, O_2_ evolution is substantially more vigorous than the bubble evolution of GC decomposition products. For the pictures displayed in (D), the iRDE tips were kept stationary to enable the clear observation of bubble generation and detachment. Results shown in (A) and (B)−(C) were obtained from independent experiments. The IrO_x_ loading was, in all cases, 50 μg_Ir_ cm^–2^ and the electrolysis conditions were *j* = 10 mA cm^–2^, *f* = 1000 rpm, Ar-saturated 0.1 M H_2_SO_4_. The bubbles observed on the anode surface continuously detach upon growth and coalescence. Under forced convection, they immediately detach from the electrode. FE_O2_ in (A) stands for the Faradaic efficiency of O_2_.

**Figure 5:**
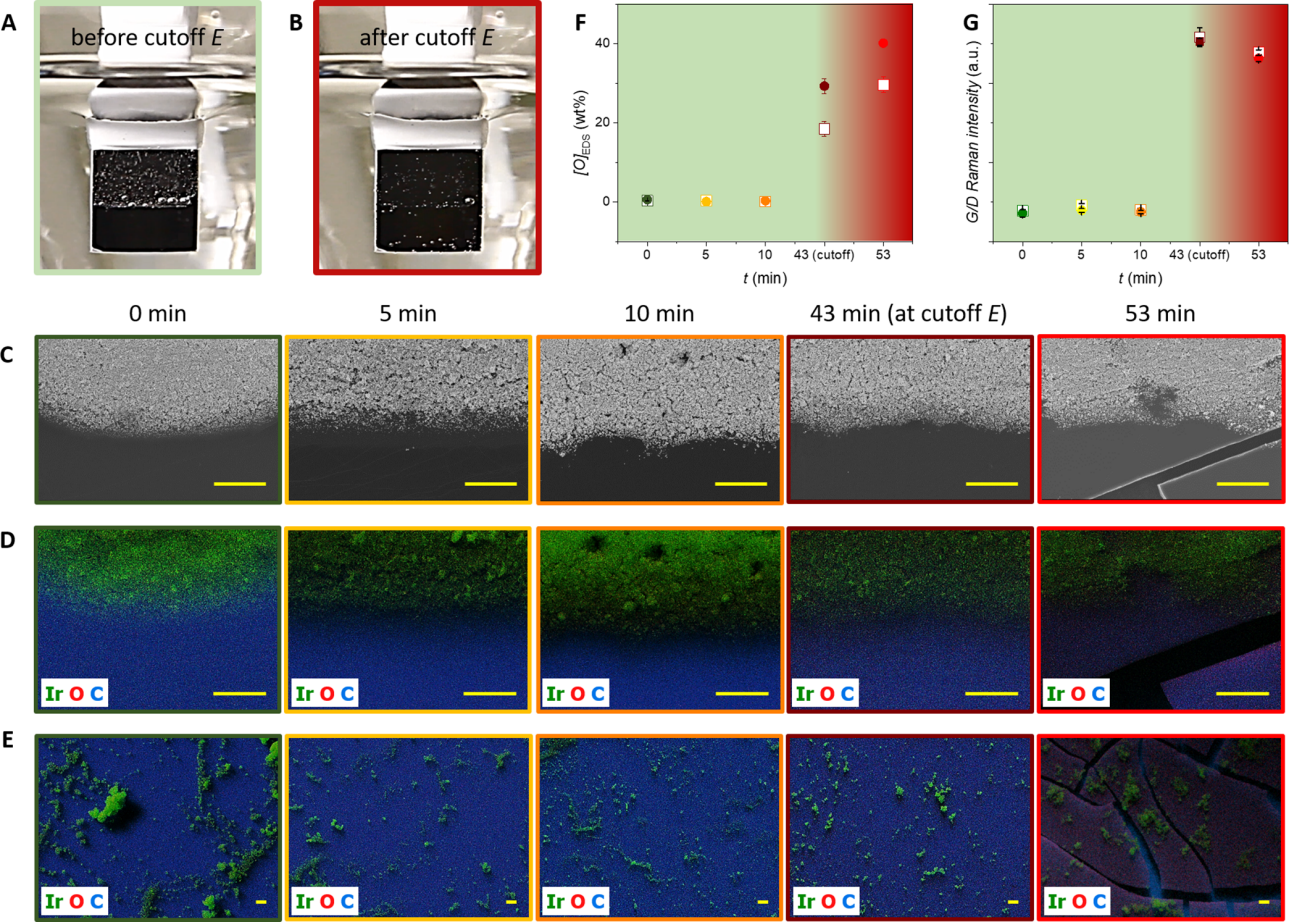
(A) and (B) Pictures of a stationary GC electrode taken before and after attainment of the cutoff potential during galvanostatic OER electrolysis. The electrode was coated with an IrO_x_ catalyst on its upper half surface only. Clearly, bubble evolution is exclusively observable on the catalyst-coated region as long as the cutoff potential does not appear. Once the potential plateaus, less vigorous bubble formation and evolution set in on both catalyst-free and catalyst-coated GC substrates. (C) and (D) SEM and EDS analyses of catalyst-rich|catalyst-free boundary layers after having undergone galvanostatic OER electrolysis for distinct times. (E) EDS analysis on catalyst-coated GC surfaces that were subjected to galvanostatic OER electrolysis as in (C) and (D). (F) EDS-derived oxygen uptake by bare GC on catalyst-free (empty squares) and catalyst-coated regions (full circles) corresponding to the analysis in (D) and (E). (G) Raman-derived G/D intensity ratios acquired on catalyst-free (empty squares) and catalyst-coated regions (full circles) from samples shown in (C)–(E). For (F)–(G), only catalyst-free surfaces were analyzed on catalyst-coated and uncoated sample regions and the error bars were extracted from measurements conducted on at least three distinct sample locations. The color code in (C)–(G) represents the applied electrolysis time. The IrO_x_ loading was, in all cases, 50 μg_Ir_ cm^–2^ (on the catalyst-functionalized half substrate surface). The electrolysis conditions were *j* = 10 mA cm^–2^ (relative to the whole immersed surface), O_2_-saturated 0.1 M H_2_SO_4_. The scale bars are 5 μm.

The results of the previous section provide screenshots of the anode’s morphology, composition, and chemical nature before and after the galvanostatic OER. Although they demonstrate the essential role of substrate failure in AMS-based OER stability screening, they do not reveal the dynamics of the degradation process occurring during the electrolysis. The understanding of this phenomenon is important since it could provide insights into how to minimize undesirable electrode assembly degradation and, in the best case, its prevention. Therefore, to gain mechanistic insights into the degradation occurring during the NP-TF-(i)RDE-based galvanostatic OER, we present herein direct monitoring of the electrolysis process by online gas chromatography. iRDE experiments using the GC-supported catalyst were coupled to headspace analysis while carrying out the OER at a specific current density *j* = 10 mA cm^–2^ (mass current density 200 A g_Ir_^–1^). Although independent experiments were performed to detect and quantify produced O_2_ and gaseous products of GC decomposition, the obtained potential transients exhibit excellent reproducibility (Figure 4A–C). Figure 4A shows that after a short latency of about 25 min, O_2_ is quantitatively generated [Faradaic efficiency (FE_O_2__) = 100%] over a period that extends as long as the exponential potential increase in the chronopotentiograms does not emerge. The initial deficit obeys the solvation of produced O_2_ in the initially deoxygenated electrolyte. Contrary to control experiments carried out with bare GC (Figure S7), Figure 4B,C shows that neither CO nor CO_2_ are detected over long periods as long as O_2_ is steadily produced on the IrO_x_-coated substrates. Then, as the galvanostatic measurements proceed, three coinciding events can be observed: (i) the onset of the abrupt potential rise, (ii) the sharp decline of the O_2_ production, and (iii) the sudden generation of CO and CO_2_. Note that the concentration of the decomposition products of GC is substantially lower than that of generated O_2_. This transition is very pronounced and even visible to the naked eye if no rotational frequency is applied (Figure 4D). These results demonstrate that the GC substrate does not decompose into gaseous CO and CO_2_ as long as the exponential potential increase does not appear. However, they neither exclude the oxygen uptake by GC at earlier stages nor provide spatial information. To address these two points, we devised the following experiments. A series of squared GC electrodes were prepared by drop-casting the IrO_x_ catalyst (50 μg_Ir_ cm^–2^) on their upper half surfaces. Both catalyst-functionalized and catalyst-free surfaces were immersed in the electrolyte and subsequently subjected to galvanostatic OER electrolysis for distinct time periods while recording the bubble evolution with a camera positioned perpendicularly to them (Figure S8A). The applied electrolysis periods included times shorter and longer than those needed to reach the cutoff potential. For reference, two more samples were added to this series: one that was not electrochemically screened and a second one whose electrolysis was stopped at the moment the potential jump plateaued (43 min). As shown in Figures 5A,B and S8B, O_2_ is the only gaseous product detected, provided that the potential does not undergo the abrupt jump, and is exclusively produced on the IrO_x_-coated region. As soon as the cutoff is reached, less vigorous bubble evolution is observed not only on the catalyst-coated region but also on the bare substrate surface, and O_2_ production drops as that one of CO and CO_2_ takes over (Supporting Information Video S3). To reveal the mechanism of oxygen uptake by GC, EDS and Raman spectroscopy characterizations were carried out on both functionalized and bare GC. Figure 5C,D displays postelectrolysis SEM-EDS analysis of the catalyst-coated|catalyst-free boundary layers. Figure 5E displays EDS analysis from the catalyst-coated regions. Oxygen content on the bare GC surfaces was extracted from the EDS elemental mappings of both catalyst-free and catalyst-containing domains (lower region in Figure 5D and catalyst-free regions in Figure 5E, respectively; see also Figure S9). Figure 5F shows the time evolution of oxygen uptake by GC during the galvanostatic OER in catalyst-free (empty squares) and catalyst-rich (full circles) regions. Regardless of the specific sample location, the bare substrate material does not oxidize, provided the electrolysis time is not extended until the sudden potential rises. Only when the cutoff threshold is reached or surpassed does the GC become passivated. Complementary Raman spectroscopy was also employed to monitor the oxygen incorporation on the GC surfaces by the distinct applied electrolysis. Raman spectra were acquired on catalyst-free GC surfaces from both functionalized and nonfunctionalized substrate locations. The corresponding G/D signal intensity ratios were extracted and are plotted in Figure 5G as a function of the applied electrolysis time. A relative increase in the G/D intensity ratio indicates a higher degree of GC oxidation (Figure S4G). Remarkably, electrodes that were subjected to short-living OER electrolysis without reaching the potential jump did not show any relative G/D intensity increment and stayed at the same level as the nonelectrolyzed sample. On the other hand, the samples that sustained the OER up to or beyond the sharp potential rise delivered a substantially larger G/D ratio. In addition, the sample that sustained electrolysis 10 min beyond the appearance of the potential plateau underwent severe structural alteration similar to the results displayed in Figure S4D. The right-most images in Figure 5C–E show that rather large fissures on the substrate surface form the boundaries of catalyst-coated passivated GC flakes. The latter are not only electrically isolated from the underlying nonoxidized bulk GC but also weakly attached to it. Figure S10 presents an EDS analysis of selected isolated flakes, showing that not only their outermost surface but also their sidewalls are fully oxidized. Three-dimensional (3D) optical microscopy analysis is also provided, showing that the substrate passivation can extend over depths close to 10 μm.

**Figure 6:**
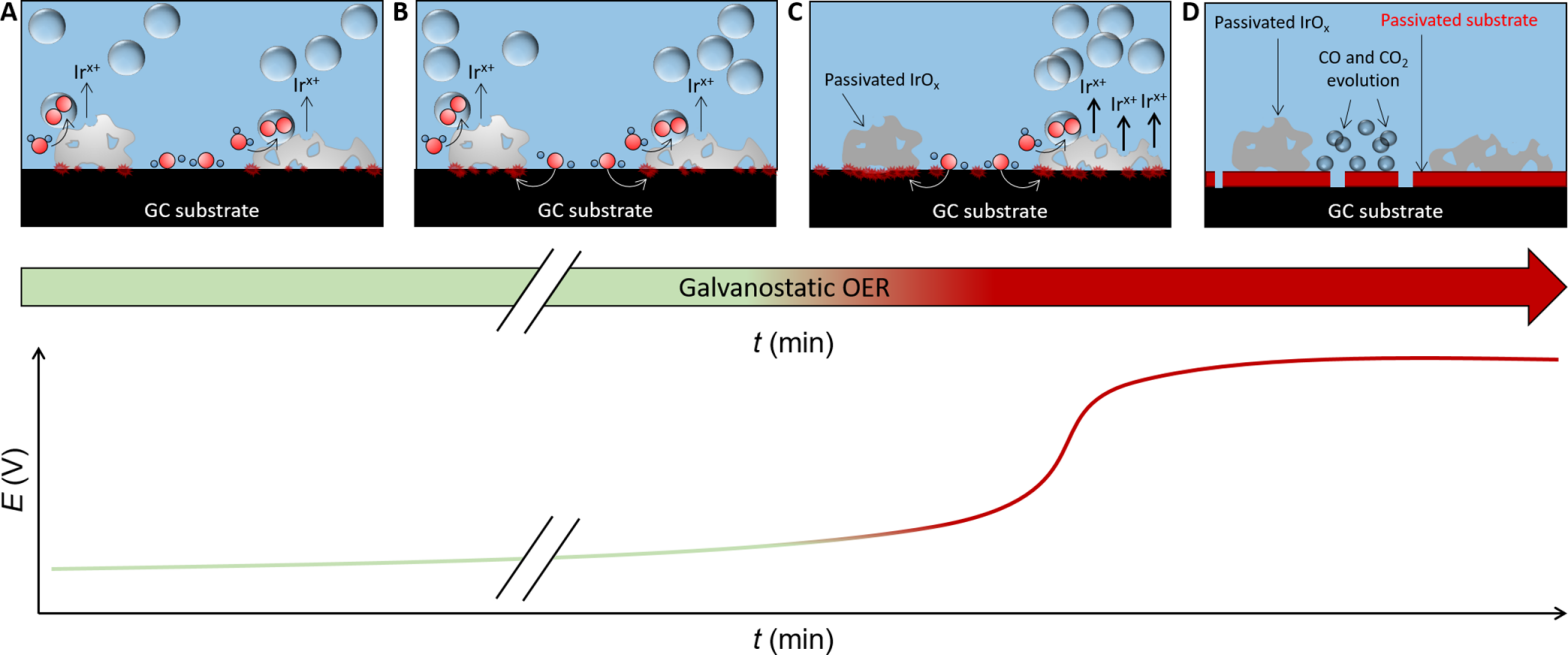
Degradation mechanism of the GC backing substrate in the course of galvanostatic OER electrocatalyst testing in NP-TF-(i)RDE-based AMS. (A) Initially and for a relatively long period, the OER is overwhelmingly kinetically favored on the surface of the catalyst material. Only minor catalyst dissolution and local GC passivation at the three-phase boundary occur (catalyst–substrate–electrolyte interface). (B) The passivated perimeter gradually grows and creeps in-between the bottom of catalyst clusters and the underlying GC. The corresponding potential transient exhibits a monotonous increase up to this stage. (C) Small catalyst clusters then become passivated through electrical detachment from the bulk GC, and the still active catalyst domains undergo harsher oxidizing conditions leading to higher local catalyst dissolution. The few remaining catalyst active sites alone cannot sustain the fixed charge flow at the electrode interface, and oxidation of bare GC between passivated catalyst islands sets in. At this stage, the potential transients undergo an exponential increase. (D) Eventually, all catalyst islands become electrically isolated due to the contact resistance imposed by the passivation of a thick substrate layer. From that moment on, the potential transient plateaus at the substrate-dependent oxidation potential and CO and CO_2_ production commences.

These findings, together with the gas evolution dynamics and the gas chromatography results presented in Figures 4, 5 and S8B, enable a mechanistic understanding of the electrode assembly degradation in NP-TF-(i)RDE-based OER AMSs. Figure 6 illustrates the key stages leading to such system failure when GC is used as a substrate material. We exclude the influence of bubbles since, as we showed in the first part of this Results section, compared to the substrate degradation, in our measurements, they do not play a significant role in the catalyst dissolution and the electrochemically derived underestimated catalyst durability. The galvanostatic OER screening imposes a fixed charge flow from Faradaic processes taking place at the solid–liquid interface. Initially, the overwhelmingly kinetically favored OER occurs exclusively on all of the IrO_x_ active sites exposed to the electrolyte (Figure 6A). The actual specific current density on the catalyst surface is higher than what is nominally set as the bare substrate material in contact with the electrolyte remains inert at this stage. The OER is accompanied by minor catalyst dissolution (Figure S11). Importantly, a small fraction of the catalyst active sites is involved in the three-phase boundary layer surrounding catalyst clusters where electrolyte, catalyst, and substrate meet. Consequently, even disregarding edge effects, the local high current density at those edges causes high local overpotentials that induce local substrate passivation.^59,60^ Thus, a thin passivated perimeter forms at all three-phase boundaries. We suggest that fissures form that might channel the electrolyte into deeper layers of the substrate. The local passivation gradually extends along the substrate underneath the porous catalyst clusters (Figure 6B). Up to this stage, this process is manifested in the potential transients through a monotonous potential increase (Figure 4A–C, green region). At some point, the growing passivation front surrounds from below the smaller catalyst clusters that become electrically isolated from the bulk GC and do not contribute from that moment on to the OER (Figure 6C). This imposes increasingly higher local current densities on the bigger catalyst islands and the three-phase boundaries that are not yet completely isolated. Eventually, only a few catalyst sites remain active that undergo substantially harsher oxidizing conditions and dissolve within a very narrow time window at higher rates (Figure S11). At that stage, they cannot account for the whole imposed charge flow and oxidation of bare GC located in-between passivated catalyst clusters becomes increasingly favored. This is the reason for the appearance of the exponential potential increase in the chronopotentiograms that was previously wrongly attributed to full intrinsic catalyst degradation or surface shielding by bubbles. When all catalyst materials on the increasingly oxidized GC become electrically isolated from it, oxidation of bulk GC sets in at the plateau potential value observed thereafter in the electrochemical measurement (Figures 6D and S8B). From that moment, CO and CO_2_ production takes over. This means that the substrate degradation shown in Figure S4 and similar reports^15,21,30^ is a sequential process comparable to a cascade reaction that occurs only after the abrupt potential jump in galvanostatic OER appears as shown in Figures 4B,C and 5F,G. The main contribution to intrinsic catalyst dissolution takes place when the cutoff potential is reached (Figure S11). This is further corroborated through a comparison of alternating potentiostatic ORR cycles measured before (Figure 3A) and after (Figure S12) attainment of the exponential jump in the galvanostatic OER. Contrary to the potentiostatic ORR cycles acquired either before starting galvanostatic OER electrolysis or during electrolysis prior to the emergence of the cutoff, those acquired after the exponential potential increase show surface-confined reductive processes that might be related to nongaseous hydroquinone-like species formed once the potential plateau is established.^21,55,61^

## Discussion and Conclusions

Our investigations aimed at elucidating the reasons leading to discrepant OER catalyst stability assessment between NP-TF-RDE-based AMS and MEA studies. By employing the iRDE approach that exploits buoyancy and anode hydrophilicity existing under operating acidic OER conditions, we successfully isolated the effects of operational substrate degradation from those related to catalyst shielding by electrogenerated bubbles. These two phenomena have been deemed the two main reasons for the apparent catalyst degradation in OER studies that lead to the overestimation of material instability in NP-TF-RDE-based AMS. The findings presented in the Results section unambiguously demonstrate that even when bubble accumulation within the catalyst layer can be avoided, the stability misalignment between galvanostatic NP-TF-RDE- and MEA-derived data is still observed and is due to the use of corrosion-prone substrate materials in AMS. Although there exists a minor effect of trapped bubbles inside a porous electrocatalyst on the signals recorded during conventional RDE-based experiments that might be of relevance for other metrics (e.g., activity, overpotential, etc.),^59^ their impact on our iRDE- and RDE-based galvanostatic OER stability studies is minor.

Results section provides mechanistic insights into anode assembly degradation induced by the galvanostatic OER. We show exemplarily for catalyst-functionalized GC-(i)RDEs that progressive passivation of the substrate material occurs exclusively at the three-phase boundary layer (catalyst–substrate–electrolyte) during galvanostatic OER electrolysis, provided the exponential increase of potential is not manifested. The local passivation likely induces crevices that channel the electrolyte inside catalyst–substrate interfaces that were initially not exposed to the electrolyte. The smaller catalyst clusters on the GC electrode are rapidly enveloped from below by a passivated GC layer and become electrically isolated from the underlying bulk GC. The still active larger catalyst clusters that sustain longer electrolysis periods without being fully passivated are increasingly subjected to higher local current densities that accelerate material dissolution. Thereafter, the few still OER active clusters cannot account for the fixed imposed charge flow, and oxidation of the bare GC surface sets in at the expense of reduced O_2_ production. This corresponds to the exponential increase in the potential observed in the chronopotentiograms. At that point in time, all catalyst material becomes isolated from the electrochemical circuit, and bulk GC oxidation takes over. The other two scrutinized substrate materials undergo slightly different degradation processes. Ti instantaneously forms poorly conducting oxides at the three-phase boundary under anodizing electrochemical conditions.^21,27^ Therefore, the very high contact resistance of the passivated surface layer that forms as soon as the catalyst clusters become fully isolated does not allow any further Faradaic process to occur. Thus, the potential overshoots at that stage and the compliance voltage of the measuring instrument is reached (Figure 2, cyan potential transients). This also explains why the potential transients obtained on this material are more short-lived than on GC and Au. When using Au, in addition to surface passivation, Au dissolution also occurs.^9,46−48^ Supporting our proposed electrode degradation mechanism, Figure S13 shows that Au dissolution indeed takes place mainly when complete isolation of the catalyst material sets in (when the cutoff potential reaches its maximum value). This explains why IrO_x_ detachment was more substantial on Au than on GC and Ti when sustaining electrolysis beyond the cutoff potential (Figure S4D–F). We propose that local Au dissolution at the contact regions between the catalyst and substrate weakens their union. The vigorous gas evolution and applied forced convection might then cause the observed partial catalyst detachment (Figure S4E).

Note that due to inefficient O_2_ bubble management in galvanostatic NP-TF-RDE testing, intermittent shielding of the catalyst layer partially prevents passivation of three-phase boundaries and induces higher local current densities on the bubble-free surfaces. This local protection periodically vanishes upon coalescence and subsequent bubble detachment. Therefore, RDE chronopotentiograms exhibit steeper potential transients than those acquired with the iRDE and show delayed emergence of the exponential increase in potential (Figures 2, 3B–D and Table S1). This explains why El-Sayed et al.^15^ could partially recover the OER activity of stressed NP-TF-RDE anodes by keeping them in the deoxygenated electrolyte at open circuit potential. In contrast, poststressed NP-TF-iRDE electrodes did not exhibit any recovery when treated likewise (Figure S14). This supports again that convective upward-facing iRDE tips completely remove electrogenerated gas bubbles.

Figure 2 shows that although higher material loadings generally lead to higher *S*_ICPMS_ and *S*_gal_, the correlation is not linear, and more importantly, the electrochemically derived stability data prove even less representative of the ICP-MS-based intrinsic catalyst degradation. This might have critical implications when trying to project results from AMS systems to technical approaches that utilize significantly larger amounts of catalyst loadings. In fact, Table S1 shows that ICP-MS-derived catalyst lifetimes in the 1–9 months range would be achieved if the ICP-MS-derived stability metrics in Figure 2 were extrapolated to the typical 2 mg_Ir_ cm^–2^ loadings applied in MEA investigations.^62^ These stability figures would still fall short of those usually observed in MEA studies. Note, however, that the increased catalyst coverage on the different substrates would substantially reduce the relative amount of corrosion-prone three-phase boundaries, which probably could not be completely enveloped from below by a passivation layer.^23^ Consequently, at these higher loadings, the gradual increase in potential would be less steep, the appearance of the cutoff value pushed back to later times, and the intrinsic catalyst degradation largely suppressed (electrochemical dissolution, Figures S11 and S13). This explains, at least to some extent, why much longer catalyst lifetimes are observed in MEA- than in AMS-based studies.

**Figure 7:**
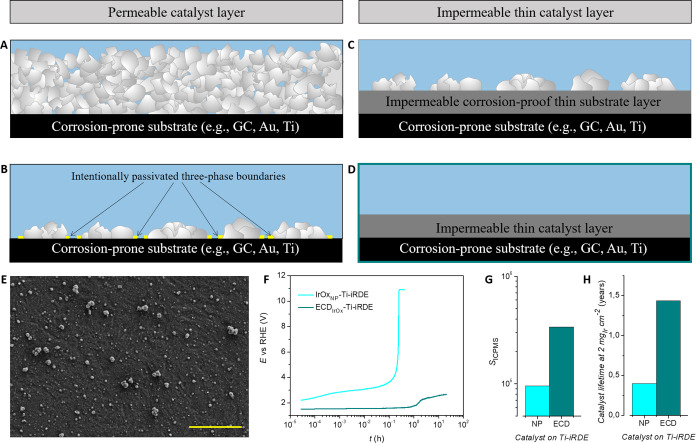
Prevention or mitigation of substrate oxidation while carrying out NP-based catalyst stability assessment in AMSs might be achieved: (A) using an excess amount of catalyst loading to limit electrolyte contact with the substrate, (B) locally passivating the boundary layers where electrolyte, catalyst, and substrate meet, and (C) introducing a corrosion-resistant conductive layer between a substrate and a catalyst. A different concept consists of utilizing compact (impermeable) catalyst layers on top of typically used substrates (e.g., GC, Au, Ti) to avoid participation of the underlying material in the electrochemical process, as depicted in (D). (E) Representative SEM analysis of ECD_IrOx_ on Ti-iRDE substrates synthesized as reported in ref (27) and Supporting Information Note 3. (F) Comparison of galvanostatic OER screening of NP-based IrO_x_ (IrOx_NP_) and ECD_IrOx_ on Ti-iRDE substrates. (G) and (H) *S*-numbers and extrapolated catalyst lifetimes at 2 mg_Ir_ cm^–2^ for IrOx_NP_ and ECD_IrOx_ on Ti-iRDE substrates. The OER electrolysis was stopped at 20 h for ECD_IrOx_-Ti-iRDE. The OER electrolysis conditions were *j* = 30 mA cm^–2^, *f* = 1000 rpm, O_2_-saturated 0.1 M H_2_SO_2_. The scale bar in (E) is 5 μm.

Since the severe catalyst morphological degradation, delamination, and dissolution shown in Figure 5, S4, and S9–S11 are produced almost exclusively after having reached the cutoff potential, strategies to avoid electrical isolation between the catalyst and the substrate should strengthen the reliability of AMS-based catalyst screenings. Yet, the fundamental reason for the substrate passivation in galvanostatic NP-TF-(i)RDE OER experiments is the permeability of powdered catalyst films that allows the formation of the three-phase boundary layer (electrolyte–catalyst–substrate). Avoiding their exposure to the electrolyte and/or developing corrosion-proof substrate materials appear to be two evident steps toward prevention of the pitfalls in current OER stability investigations carried out through AMS approaches. These two concepts are illustrated in Figure 7. Three ways to hinder physical contact between substrate materials and the electrolyte using NP-based OER catalysts are depicted in Figure 7A–C. Similarly to what occurs in MEAs, large loadings of powdered catalysts on noninert substrates limit the mass transport of electrolytes to the corrosion-prone surface, thus significantly reducing the rate of substrate surface oxidation (Figure 7A).^5,23^ Another alternative solution to the degradation of the catalyst–substrate interface could be achieved through its protection prior to electrochemical operation (Figure 7B). For instance, it has been shown that local electron beam irradiation on nanomaterials synthesized by additive-assisted colloidal methods can lead to their improved structural stability through the transformation of the adsorbed surfactants into dense carbonaceous shells.^43,63,64^ Rutile IrO_2_ is *per se*, almost corrosion- and passivation-proof even under very harsh anodic conditions. Recently, Zlatar et al. proposed the functionalization of electrodes with IrO_2_ thin-film layers prepared by physical vapor deposition (PVD) on which non-noble metal powdered catalysts could be drop-casted (Figure 7C).^9^ This would reliably enable intrinsic catalyst stability assessment of the less noble material by ICP-MS-based metrics (e.g., *S*-number, ASF-Factor). These authors suggest that minor dissolution of the IrO_2_ substrate under water oxidation conditions is well understood and can be accounted for.

A different concept that does not employ powdered catalyst materials is depicted in Figure 7D. It utilizes compact thin-film catalyst layers (non-NP-based) and prevents physical contact between oxidizing species and the underlying substrate. Ideally, pinhole-free vacuum-based or electrochemically deposited (ECD) Ir or IrO_x_ layers can be implemented on (i)RDE substrates as it has been done on Ti PTLs by Carmo et al.^37,65^ and Choe et al.^27^ As a proof of concept, we present in Figure 7E,F morphological characterization and galvanostatic OER catalyst stability screening of Ti-iRDE electrodes that were functionalized with ECD thin IrO_x_ layers (ECD_IrOx_-Ti-iRDE, see Supporting Information Note 3 and Table S1). Figure 7F compares the performance of this quasi-impermeable catalyst to that of the nanoparticulate IrO_x_ catalyst on Ti-iRDEs (IrOx_NP_-Ti-iRDE, 50 μg_Ir_ cm^–2^, cyan curve in Figure 2J). Unlike the IrOx_NP_-Ti-iRDE sample that withstands the OER electrolysis for only a few minutes, the ECD_IrOx_-Ti-iRDE sample sustains it for much longer periods under the same conditions without losing activity or undergoing isolation from the substrate (the electrolysis was stopped after 20 h). In addition, the *S*-number and catalyst lifetime of the electrodeposited material showcased improved stability compared to those corresponding to the porous NP-based catalyst (Figure 7G,H and Table S1). Although the potential transient of the ECD_IrOx_-Ti-iRDE sample underwent a gradual increase (see Supporting Information Note 3), its long-term durability proves that this approach consisting of avoidance of electrolyte permeation to the (i)RDE substrate material brings us closer to artifact-free intrinsic catalyst stability assessment by AMS studies. As explained in Supporting Information Note 3, the eventual degradation of this prototype ECD_IrOx_-Ti-RDE assembly is rooted in the existence of locally discontinuous sample regions that are prone to passivation (although at a much lower rate than for IrOx_NP_-Ti-iRDE, Figure S15). We suggest that further optimized deposition conditions will enable pinhole-free IrO_x_ catalyst layers with outstanding stabilities and lower material loading. Importantly, these electrochemically deposited catalyst materials are transferable to Ti PTLs used in MEAs and actual electrolyzers and would allow more reliable projection of stability metrics to technical platforms than current powdered-based AMSs.^37,65^ Moreover, Ti PTLs functionalized this way would not require additional protecting coatings with noble metals typically used in MEA assemblies.^13,37^ Implementation and testing of this or other local protection strategies (Figure 7A–D) would contribute to the development of advanced interfacing between Ti PTLs and CCMs in MEAs.

Finally, we advocate for the development of stability approaches coupling iRDE settings to improved substrate–catalyst interfacing, as proposed above. This strategy should enable reliable catalyst degradation evaluation and a mechanistic understanding of intrinsic catalyst stability.^66^ Standardized protocols relying on these experimental platforms that tackle the issues encountered in typical ink-casted and nanoparticle-based TF-RDE experiments should be fostered, developed, and widely implemented.^67^ Reliable and fair comparisons between these investigations and MEA-based stability studies should allow better transferability to technical approaches and boost PEMWE development. However, it is important to acknowledge that in MEA settings, there are additional catalyst dissolution pathways compared to the iRDE-based approach, namely, through the membrane separating the anode and the cathode.^68^ Complementary approaches based on gas diffusion electrodes that enable the mass balance of dissolved catalyst material in more realistic settings should also be resorted to.^69,70^ Nevertheless, we are certain that the (i)RDE technique will continue playing an essential role in experimental electrocatalysis in future research directions.^26^

## Supporting Information Available

Morphological and compositional characterization of powdered IrO_x_-(i)RDE anodes before and after galvanostatic OER; online gas chromatography analysis during the galvanostatic OER on bare GC-iRDE samples; schematics of the approach for bubble dynamics visualization at electrified GC anodes; EDS-based oxygen uptake by GC as a function of applied electrolysis time; time evolution of Ir and Au dissolution by the ICP-MS during the OER; cyclic voltammetry of reductive processes on the surface of IrO_x_-RDE anodes before and after attainment of the cutoff potential; and post-OER recovery test of screened IrO_x_-coated iRDEs (PDF)
Video S1 exemplarily shows that even in the absence of forced convection, the produced oxygen readily detaches from the catalyst-coated iRDE surface (MP4).
Video S2 shows that the bubble management achieved with the iRDE is so effective that it also induces the removal of bubbles occasionally adhering to the hydrophobic electrode shroud (MP4).
Video S3 shows that bubble evolution is observed not only on the catalyst-coated region but also on the bare substrate surface when the potential plateau appears for the GC electrodes presented in Figure 5A−B (MP4).
