# Supplementary material for: Disentangling the Pitfalls of Rotating Disk Electrode-Based OER Stability Assessment: Bubble Blockage or Substrate Passivation?
Source: ACS Catal. 2024 Nov 13;14(23):17331–46. doi: 10.1021/acscatal.4c05447 (PMC11629296; doi:10.1021/acscatal.4c05447)
Supplement: Supplementary file 1 — cs4c05447_si_001.pdf [file cs4c05447_si_001.pdf]

Disentangling the pitfalls of rotating disk electrode-based OER stability assessment: bubble blockage or substrate passivation?

Aline Bornet,[1,‡] *Pavel Moreno-García,*[1,‡,]*, *Abhijit Dutta*[1], *Ying Kong,*[1] *Mike Liechti*[1], *Soma Vesztergom*[1,2], *Matthias Arenz*[1], *Peter Broekmann*[1,]*

[1]Department of Chemistry, Biochemistry and Pharmaceutical Sciences, University of Bern, Freiestrasse 3, Bern 3012, Switzerland

[2]MTA–ELTE Momentum Interfacial Electrochemistry Research Group, Eötvös Loránd University, Pázmány Péter sétány 1/A, Budapest 1117, Hungary

‡ these authors contributed equally to the work

*pavel.moreno@unibe.ch, peter.broekmann@unibe.ch

Supplementary Information

Contents

Experimental setup for electrochemical deposition of ECD-IrO_x layers on Ti-RDEs ….…………………….2

Supplementary Note 1 ……………………….……….......…………………..…………………………….3

Supplementary Note 2 ……………………….……….......…………………..…………………………….4

Supplementary Table 1 ………..………...………………………..5

Potential transient of bare Au-RDE subjected to galvanostatic OER ………………………………..……6

Morphological and compositional transitions of IrO_x-(i)RDE anodes induced by galvanostatic OER ……...7

Time evolution of gaseous products in the course of galvanostatic OER on bare GC-iRDE ....………..…10

Visualization approach for assessment of bubble dynamics at electrified GC anodes ……….……………..11

EDS analysis of oxygen uptake by GC as a function of applied galvanostatic OER ……………………….12

EDS and 3D microscopy analysis of GC passivation and exfoliation after sustained electrolysis 13

Evolution of Ir dissolution in the course of galvanostatic GC-RDE-based OER …………...………………..14

Reductive processes on the surface of IrO_x-RDE anodes before and after the cutoff potential ……………..15

Evolution of Ir and Au dissolution in the course of galvanostatic Au-RDE-based OER ……….....………..16

Post-OER recovery test of electrolyzed IrO_x-coated iRDE anodes ………………………………………..17

Supplementary Note 3 ……………………………………………………………………………………..18

References ……………………………….......……………………………………….………………………..19

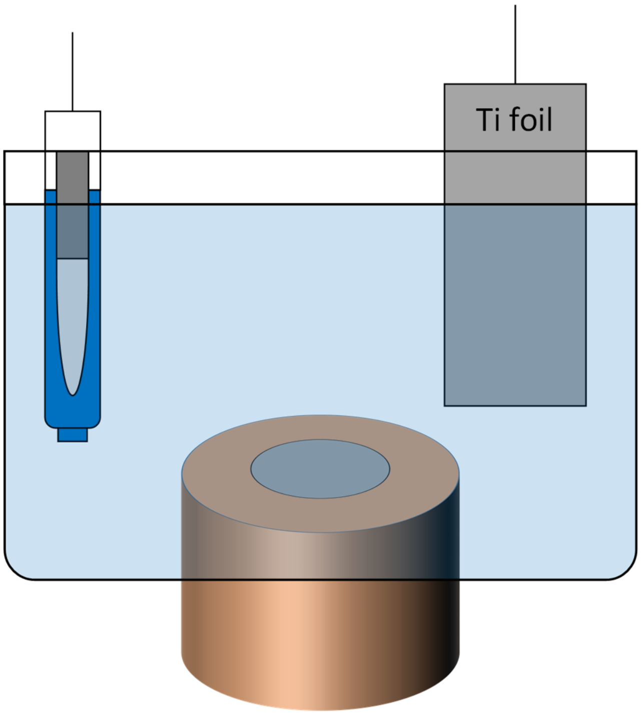

Figure S1 Experimental setup for electrochemical deposition of ECD_{IrOx} layer on Ti-RDE electrodes. The RDE tips were inserted facing upwards inside the electrochemical cell. A calomel reference electrode and a Ti foil counter electrode were used to carry out potentiostatic IrO_x electrodeposition on Ti according to the synthesis protocol of reference 1.

Supplementary Note 1

Hermetically sealed iRDE setup coupled to gas chromatography for quantitative analysis of gas evolving processes. Among hydrodynamic methods involving convective mass transport of reactants to a catalytically active surface, the RDE might be the most useful and widely employed technique in fundamental science. This is due to its ability to control the supply of reactive species at an electrode surface enabling distinction between mass transport and kinetic control in surface reactions. However, numerous relevant electrochemical processes investigated by RDE methods involve gas evolution of intermediates or products whose quantitative analysis is complicated by the fact that such studies require hermetic coupling across rotating elements, the hosting cell body and gas analysis techniques. In addition, electrochemically generated gas bubbles typically adhere to the surface of both the RDE working electrode and its embedding shaft preventing them from reaching the solution-gas interface. This partial shielding of electrode's active sites by bubble retention at the RDE tip undermines the accuracy of the electrochemical measurements and hinders quantitative analysis of the gaseous products collected from the headspace of an electrochemical cell. This challenge has been solved by employing a reconfigured inverted RDE instrument that enables the detachment of electrogenerated bubbles from the working electrode due to buoyancy and forced convection.[2-5] A schematic view and a demonstrative image of the instrument under operation are shown in **Figure S2**.

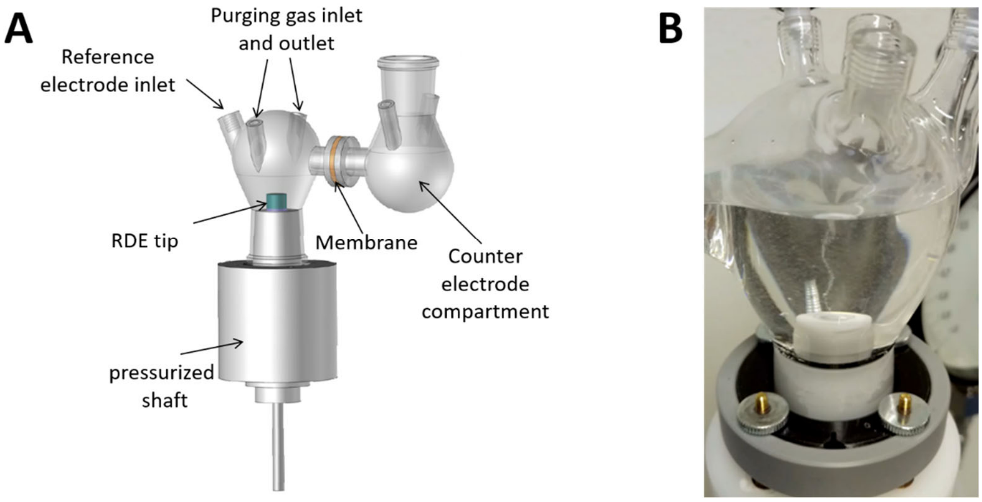

Figure S2 iRDE setup for quantitative analysis of electrochemical gas evolving processes. (A) Schematic representation of the iRDE instrument developed by our group (taken and modified from reference 3 with permission from the editor). (B) Illustration of the iRDE under operating conditions showing complete detachment of gaseous products from the working electrode surface (taken from reference 4 with permission from the editor).

Supplementary Note 2

iRDE for prevention of catalyst shielding in NP-TF-RDE studies by electrogenerated oxygen. The RDE is an inexpensive and readily accessible electroanalytical tool commonly used to study the kinetics of electrocatalytic reactions.[6] In an attempt to establish an OER catalyst stability benchmarking protocol, the Joint Center for Artificial Photosynthesis (JCAP) introduced an RDE-based stability approach.[7] However, the catalyst stability figures extracted from these AMS-based studies have recently been found to underestimate the material's stability and differ substantially from the much longer durability figures extracted from MEA approaches. El-Sayed et al. have comprehensively investigated this experimental misalliance and advocated for the inadequacy of catalyst stabilities extracted when OER investigations are carried out through NP-TF-RDE-based AMS settings.[8-10] Through rigorous electrochemical analysis, the authors suggest that the electrochemistry-derived stability metrics in AMS are not reliable because oxygen microbubbles unavoidably accumulate within the catalyst layer and gradually block the surface of the catalyst material. In this scenario, the complete blocking of the catalyst surface is manifested in the galvanostatic OER by the achievement of a cutoff potential that is mistakenly assigned to the catalyst's end-of-life. The key experimental evidence supporting their hypothesis is the nonappearance of the typically observed monotonous potential increase when the bubbles are removed from an O_2-evolving polycrystalline Ir disk electrode by an external ultrasonic field.[10] Thus, they concluded that the NP-TF-RDE approach would be suitable for OER studies only if complete gas removal from the catalyst surface is ensured. Interestingly, detachment of O_2 bubbles from gas-evolving RDE anodes proceeds, in principle, at desirable hydrophilic surfaces under typically applied OER acidic conditions. First, removal of macroscopic bubbles that extend beyond the diffusion layer is assisted by the applied forced convection.[11] Second, there are repulsive electrostatic forces between positively charged OER anodes and positively charged gas bubbles in electrolytes with $pH < 2$.[12-13] Moreover, when conducting water oxidation at high current densities, the interfacial saturation of dissolved gas can be reduced by bubble-induced micro and macroconvection that mixes electrolyte near the electrode with that in the bulk and slows down the expansion of the diffusion layer.[14-15] However, all these factors are not fully exploited when conventional NP-TF-RDE experiments are conducted because the buoyancy exerted on the formed gas bubbles by the electrolyte redirects them, at least to some extent, back to the anode surface. In addition, the trapping of oxygen bubbles in front of the catalyst layer prevents direct quantitative analysis of the product by gas analytical techniques. This is an essential drawback of RDE-based gas-evolving experiments concealing potential side reactions that may occur during OER catalyst screening (e.g., substrate oxidation, material dissolution, capacitive current effects, oxidation of adventitious species, etc.).[16-18]

Table S1 Experimental conditions of galvanostatic OER experiments and obtained catalyst stability data

Approach	Substrate	Catalyst loading / μg_{Ir} cm^{-2}	Applied specific current density / mA cm^{-2}	Applied mass current density / A mg_{Ir}^{-1}	Elapsed time until cutoff *E* / s	$E_{initial}$ / V	$E_{plateau}$ value / V	Dissolved Ir (n_{Ir}) / mol	Fraction of dissolved Ir / %	Evolved O_2[a] (n_{O2}) / mol	S_{ICPMS}	S_{gal}[b]	S_{ICPMS}/S_{gal}	Catalyst lifetime / days	Catalyst lifetime at 2 mg_{Ir} cm^{-2} / years
RDE	GC	10	30	3.00	497	1.70	2.42	2.0×10^{-10}	1.99	7.59×10^{-6}	3.74×10^{4}	7.43×10^{2}	5.03×10^{1}	0.29	0.16
RDE	GC	50	30	0.60	7828	1.63	2.39	6.8×10^{-10}	1.33	1.19×10^{-4}	1.76×10^{5}	2.34×10^{3}	7.52×10^{1}	6.81	0.75
RDE	Au	10	30	3.00	1052	1.71	2.12	5.3×10^{-10}	5.94	1.39×10^{-5}	2.65×10^{4}	1.57×10^{3}	1.68×10^{1}	0.21	0.11
RDE	Au	50	30	3.00	8204	1.68	2.12	1.7×10^{-9}	3.4	1.25×10^{-4}	7.22×10^{4}	2.45×10^{3}	2.94×10^{1}	2.79	0.31
RDE	Ti	10	30	3.00	437	4.23	compliance	1.1×10^{-10}	1.08	6.67×10^{-6}	6.07×10^{4}	6.53×10^{2}	9.29×10^{1}	0.47	0.26
RDE	Ti	50	30	3.00	4534	3.05	compliance	8.7×10^{-10}	1.71	6.92×10^{-5}	7.93×10^{4}	1.35×10^{3}	5.85×10^{1}	3.07	0.34
iRDE	GC	10	30	0.60	200	1.67	2.32	1.4×10^{-10}	1.39	3.05×10^{-6}	2.15×10^{4}	2.99×10^{2}	7.19×10^{1}	0.17	0.09
iRDE	GC	50	30	0.60	3623	1.63	2.33	3.7×10^{-10}	0.73	5.53×10^{-5}	1.49×10^{5}	1.08×10^{3}	1.37×10^{2}	5.76	0.63
iRDE	Au	10	30	0.60	344	1.71	2.10	2.2×10^{-10}	2.18	5.25×10^{-6}	2.36×10^{4}	5.14×10^{2}	4.59×10^{1}	0.18	0.10
iRDE	Au	50	30	0.60	6418	1.66	2.10	9.1×10^{-10}	2.05	8.51×10^{-5}	9.34×10^{4}	1.92×10^{3}	4.87×10^{1}	3.62	0.40
iRDE	Ti	10	30	0.60	173	4.47	compliance	6.2×10^{-11}	0.61	2.63×10^{-6}	4.26×10^{4}	2.58×10^{2}	1.65×10^{2}	0.33	0.18
iRDE[c]	Ti	50	30	0.60	906	3.03	compliance	1.4×10^{-10}	0.28	1.38×10^{-5}	9.53×10^{4}	2.71×10^{2}	3.52×10^{2}	3.69	0.40
iRDE[d]	ECD_{IrOx}-Ti	1144.2	30	2.62×10^{-2}	Stopped at 7.2×10^{4}	1.55	NA	3.2×10^{-9}	0.28	1.10×10^{-3}	3.38×10^{5}	NA	NA	299.30	1.43

[a]Derived from cumulative Faradaic charge between the starting of the measurements and the time at which the potential plateaus commence

[b]Assuming all the catalyst loaded on the employed (i)RDE tips was consumed at the moment the potentials attained the substrate-dependent plateaus

[c]This row corresponds to the results of the IrO_x NP material on Ti-iRDE shown in **Figure 2J-L** in the main text.

[d]This row corresponds to the results of the electrochemically deposited IrO_x layer on Ti-iRDE substrates (see **Figure 8F-H**, dark cyan data points).

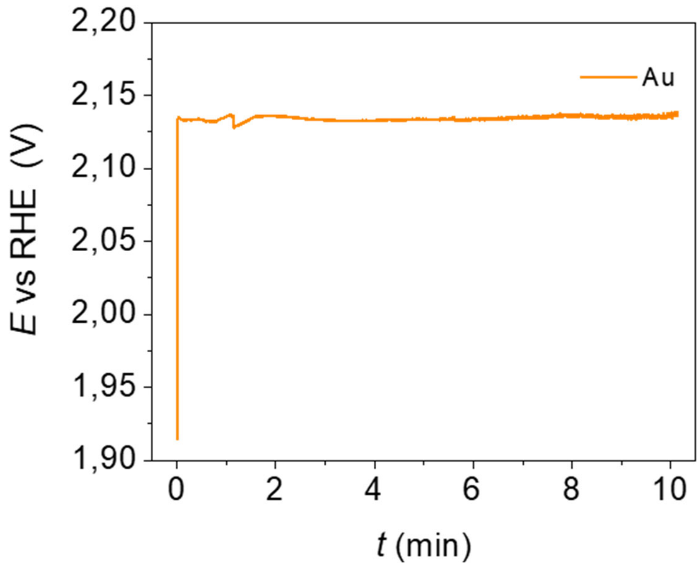

Figure S3 Potential transient of bare Au RDE electrode subjected to galvanostatic OER. After surface charging, the potential transient exhibits an electrode material-dependent plateau. The experiment was carried out at a specific current density *j* = 30 mA cm^{-2} and rotational frequency *f* = 1000 rpm in O_2-saturated 0.1 M H_2SO_4 supporting electrolyte.

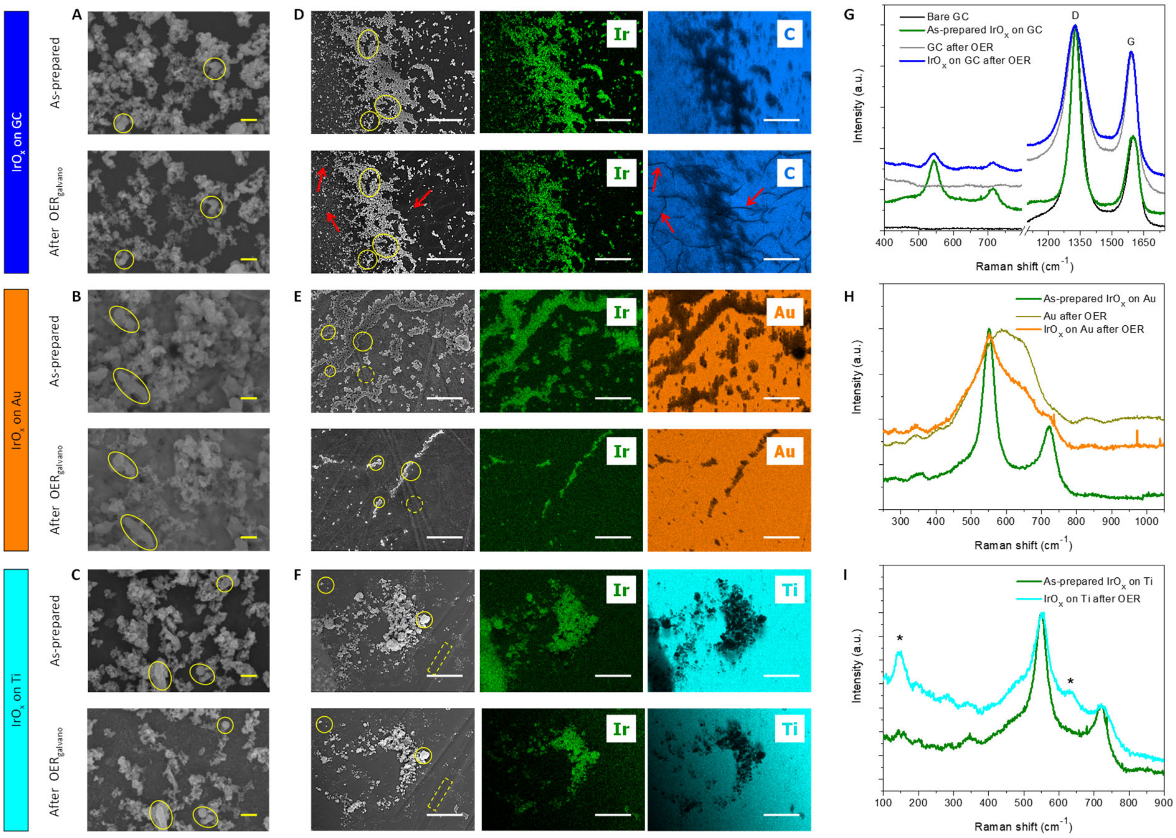

Figure S4 Morphological, compositional and chemical transitions of NP IrO_x-coated iRDE anodes induced by galvanostatic OER stability screening. (A)-(C) High-resolution IL-SEM analysis of IrO_x electrocatalyst on GC-, Au- and Ti-iRDE electrodes. The catalyst material undergoes only minor structural degradation on the three backing electrodes. (D)-(F) IL-SEM-EDS analysis on the surface of the three catalyst-coated substrates. The solid and dotted yellow shapes in (A)-(F) frame selected features of catalyst and substrate material, respectively, that are readily recognizable before and after the OER electrolyses. The red arrows point at crevices on the GC substrate that appear after OER stressing. (G)-(I) Averaged Raman spectra acquired on at least four distinct locations of catalyst-coated iRDE tips before and after OER. The signal intensity was normalized to its maximum value in each relevant range. The asterisks in (I) correspond to Raman fingerprints of anatase TiO_2. The applied electrolyses were stopped few minutes after the substrate-dependent potential plateau appeared. The IrO_x loading was in all cases 50 μg_{Ir} cm^{-2}, the electrolysis conditions were j = 30 mA cm^{-2}, f = 1000 rpm, O_2-saturated 0.1 M H_2SO_4. The yellow and white scale bars represent 100 nm respectively, 25 µm.

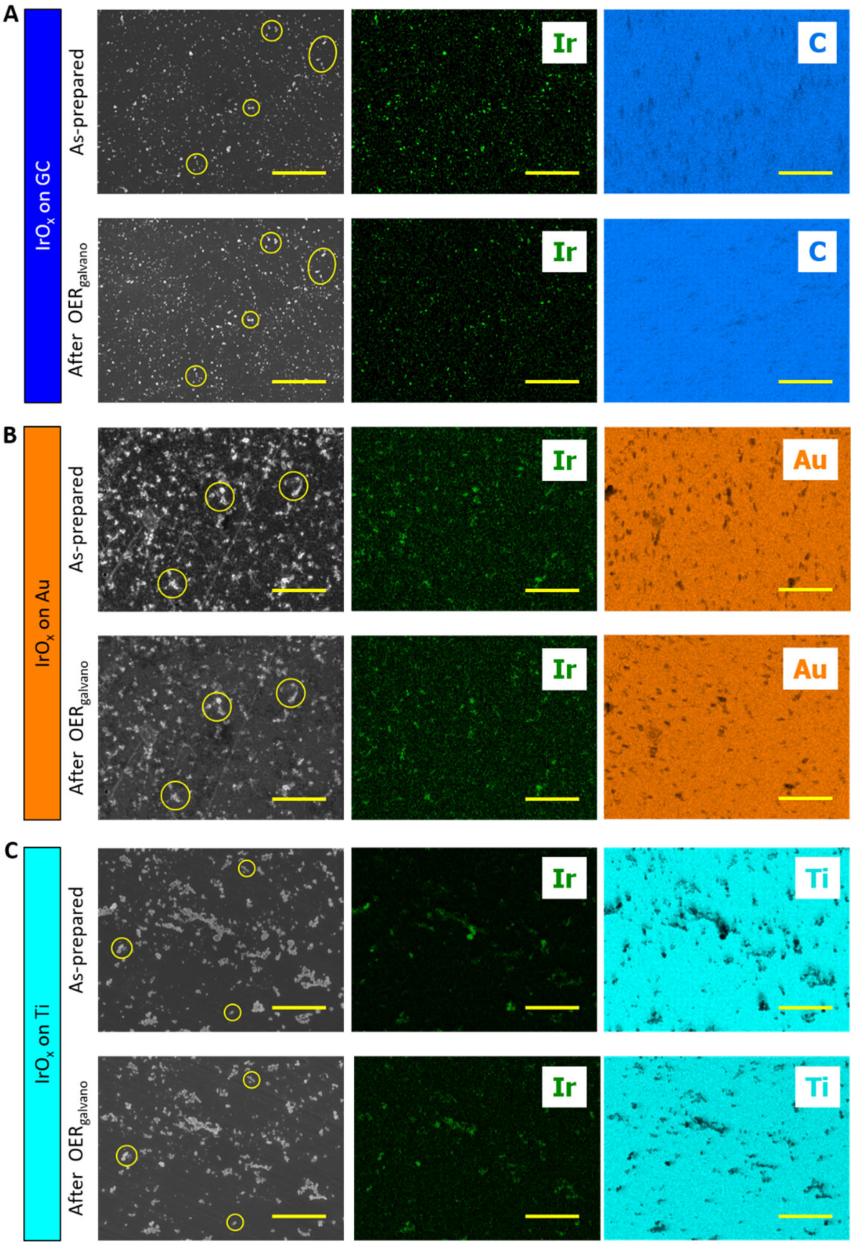

Figure S5 Morphological and compositional transitions of NP IrO_x-coated iRDE anodes induced by galvanostatic OER stability screening. (A)-(C) IL-SEM-EDS analysis on the surface of IrO_x-coated GC-, Au- and Ti-iRDE electrodes. The catalyst material undergoes only minor structural degradation on the three backing electrodes. The solid yellow shapes frame selected features of catalyst material that are readily recognizable before and after the OER electrolyses. The applied electrolyses were stopped few minutes after the substrate dependent potential plateau appeared. The IrO_x loading was in all cases 10 μg_{Ir} cm^{-2}, the electrolysis conditions were j = 30 mA cm^{-2}, f = 1000 rpm, O_2-saturated 0.1 M H_2SO_4. The scale bars represent 25 µm.

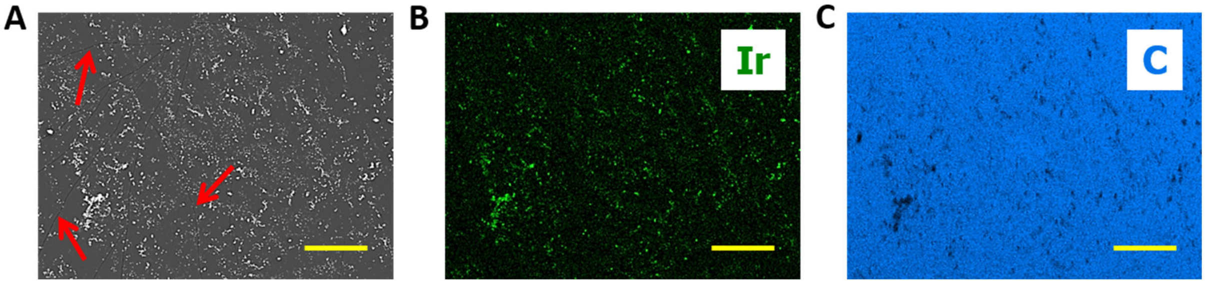

Figure S6 Post-electrolysis morphological and compositional characterization of NP IrO_x-coated RDE anode subjected to ORR-OER alternating cycling. (A) and (B)-(C) Post-electrolysis SEM and EDS analysis on the surface of IrO_x-coated GC-RDE. A substantial amount of catalyst material remains on the GC substrates after the electrolysis. The details of the applied electrolysis are described in **Figure 3** in the main text and the corresponding discussion. The IrO_x loading was 50 μg_{Ir} cm^{-2} and the electrolysis was carried out at f = 150 rpm in O_2-saturated 0.1 M H_2SO_4 supporting electrolyte. The scale bars represent 100 µm. The red arrows point at crevices on the GC substrate that appear after OER stressing.

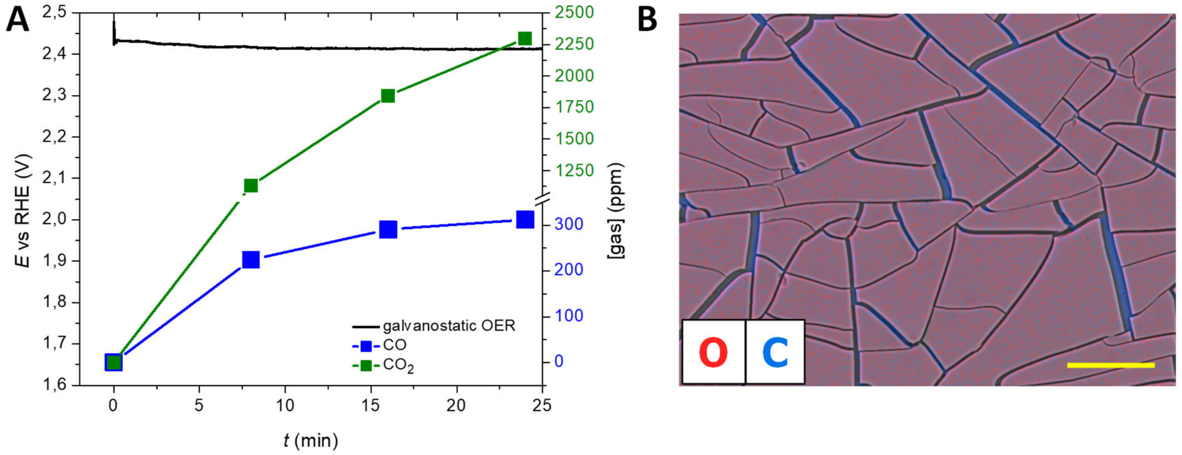

Figure S7. Time evolution of gaseous products generated in the course of galvanostatic OER experiments on a stationary bare GC electrode and resulting morphological degradation. (A) Online detection of CO (blue) and CO_2 (green) by gas chromatography during OER screening (black curve) on bare GC. (B) Corresponding post-electrolysis EDS analysis. CO and CO_2 are detected almost immediately after having started the galvanostatic OER. The first detection event was initiated 7 min after having started the water oxidation. The electrolysis conditions were j = 10 mA cm^{-2} (relative to the whole immersed surface), t = 25 min in 0.1 M H_2SO_4 electrolyte. Ar was used as carrier gas for gas chromatography analysis. The scale bar in (B) represents 100 µm.

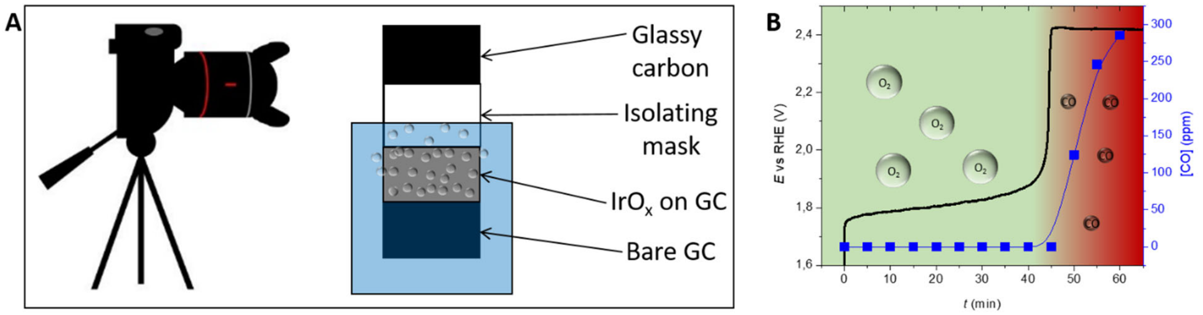

Figure S8 Schematic representation of the visualization approach for assessment of bubble dynamics at electrified half-catalyst-covered GC anodes. (A) A series of GC electrodes were prepared by dropcasting IrO_x catalyst on their upper half surfaces. Both catalyst-functionalized and catalyst-free surfaces were immersed in the electrolyte and subsequently subjected to galvanostatic OER electrolysis for distinct time periods while recording the bubble evolution with a camera positioned perpendicularly to them. (B) Online detection of CO (blue data points) by gas chromatography during galvanostatic OER screening on half-catalyst-covered GC. In this particular experiment, the electrolysis was stopped 20 min after having attained the cutoff potential. O_2 is the only gaseous product detected provided that the potential does not undergo the abrupt jump and is exclusively produced on the IrO_x-coated region. As soon as the cutoff is reached, less vigorous bubble evolution is observed not only on the catalyst-coated region but also all over the electrode surface and O_2 production drops as that one of CO and CO_2 takes over (**Supplementary Video 3**). Applied electrolysis conditions: 50 μg_{Ir} cm^{-2} catalyst loading (on half of the exposed surface), 10 mA cm^{-2} (relative to the whole immersed surface), 0.1 M H_2SO_4.

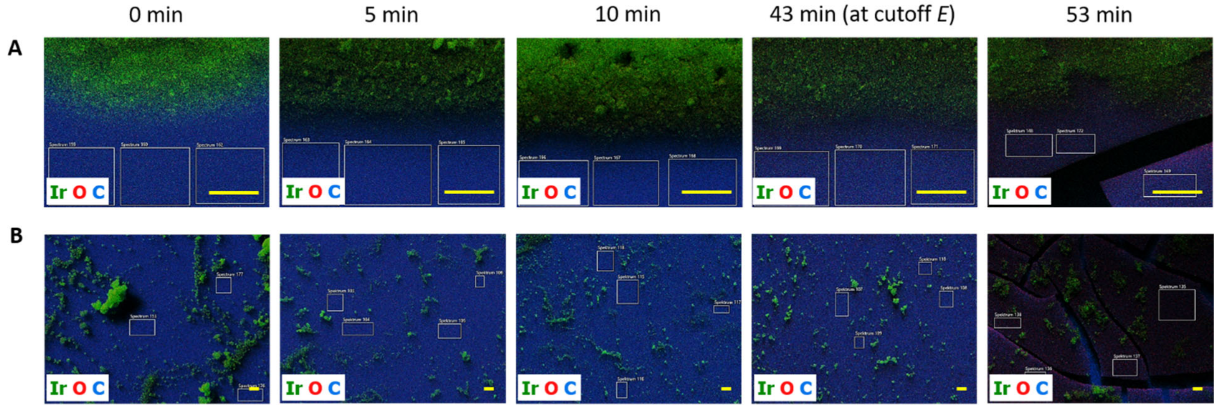

Figure S9 Oxygen uptake by GC as a function of applied galvanostatic OER. (A) and (B) EDS analysis of catalyst-rich|catalyst-free GC interfaces and catalyst-coated GC surfaces, respectively, after having undergone galvanostatic OER electrolysis for distinct times. The extracted oxygen concentrations were used to build **Figure 5F** in the main text. Experimental conditions: 50 μg_{Ir} cm^{-2} catalyst loading (on half of the exposed surface), j = 10 mA cm^{-2} (relative to the whole immersed surface), 0.1 M H_2SO_4. The scale bars represent 5 µm.

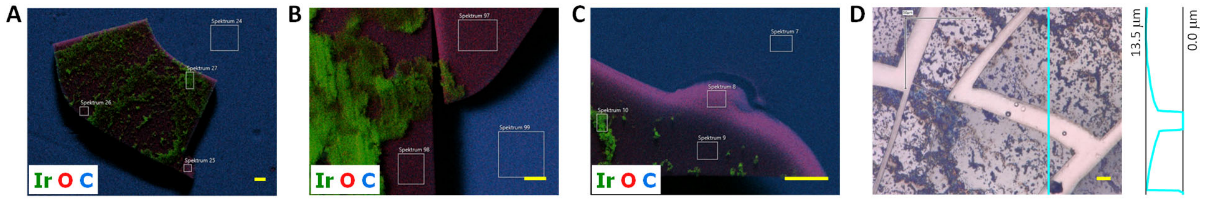

Figure S10 Extent of GC passivation and exfoliation after sustained galvanostatic electrolysis beyond the cutoff potential on IrO_x-coated substrates. (A)-(C) Post-electrolysis EDS elemental mapping of a sample prepared as shown in **Figure S8**. The electrolysis was conducted for 38 min beyond the emergence of the cutoff potential. The passivated GC layer was partially detached from the underlying non-oxidized bulk material. (D) 3D optical micrograph and depth profile along the cyan line. Experimental conditions: 50 μg_{Ir} cm^{-2} catalyst loading (on half of the exposed surface), 10 mA cm^{-2} (relative to the whole immersed surface), 0.1 M H_2SO_4. The scale bars represent 10 µm.

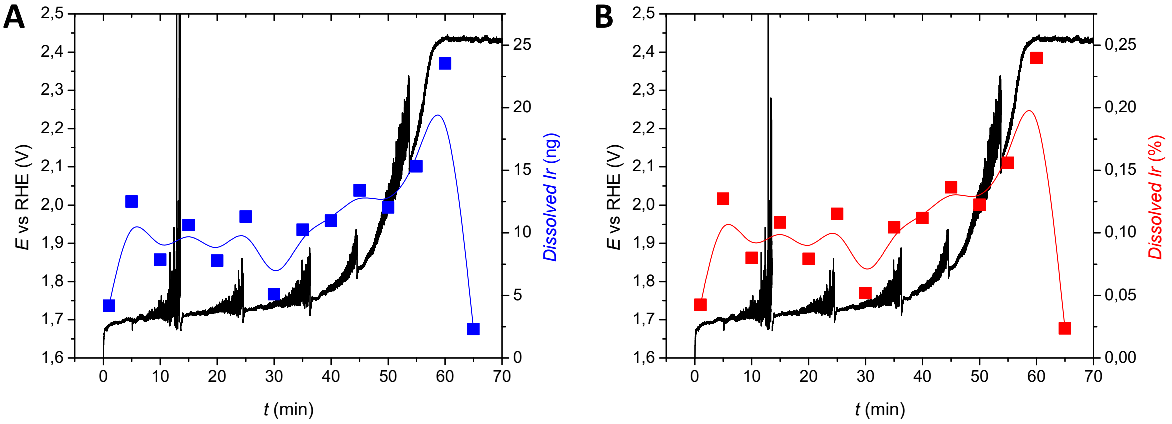

Figure S11 Non-cumulative evolution of Ir dissolution in the course of galvanostatic IrO_x-coated GC-RDE-based OER. (A) ICP-MS-derived loss of Ir mass during OER electrolysis on IrO_x-coated GC-RDE anode. (B) Corresponding loss of Ir relative to the initial amount of catalyst drop-casted on the RDE electrode. Electrolyte analyte was taken every 5 minutes from the electrochemical cell and analyzed postmortem. The concentration of dissolved Ir at any given time was calculated after having subtracted the cumulative Ir dissolved prior to it. After having reached the potential plateau, the catalyst dissolution was non-detectable. The oscillations observed in the potential transient are due to accumulation and release of the bubble cloud that builds in front of the RDE anode. Applied electrolysis conditions: 50 μg_{Ir} cm^{-2}, 30 mA cm^{-2} and f = 1000 rpm in 0.1 M H_2SO_4 supporting electrolyte.

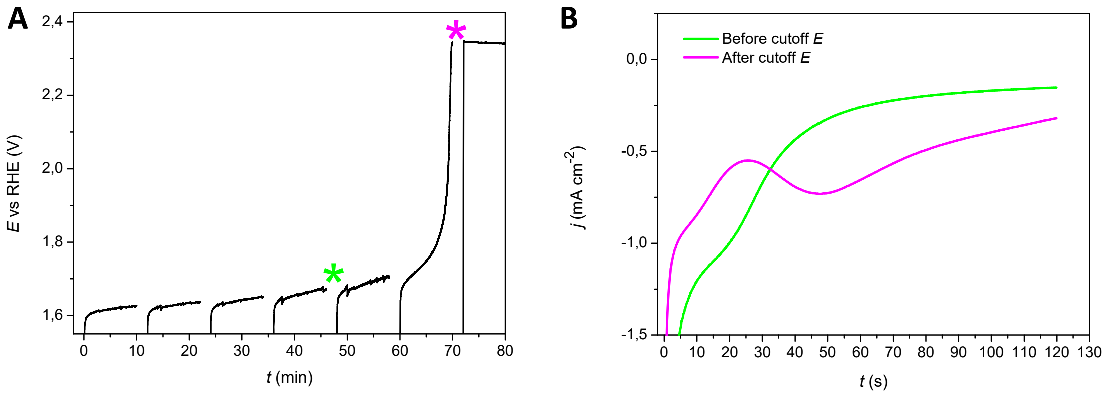

Figure S12 Reductive processes on the surface of a downward-facing IrO_x-RDE anode before and after reaching the cutoff potential in galvanostatic OER. (A)-(B) Alternating 10-min galvanostatic OER cycles (10 mA cm^{-2}) and 2-min potentiostatic ORR steps (0.2 V vs RHE) were applied. Potentiostatic ORR cycles recorded before attainment of the abrupt potential jump in galvanostatic OER show reduction of trapped bubbles inside the catalyst layer of IrO_x-TF-RDE anodes (green line in (B)). After the appearance of the plateau value in galvanostatic OER, subsequently recorded ORR potentiostatic cycles display reductive processes of the oxidized GC substrate (pink line in (B)). The asterisks in (A) show selected intervals at which potentiostatic steps (B) were applied between galvanostatic OER cycles. Electrolysis carried out at 150 rpm in O_2-saturated 0.1 M H_2SO_4 supporting electrolyte, The catalyst loading was 50 μg_{Ir} cm^{-2}.

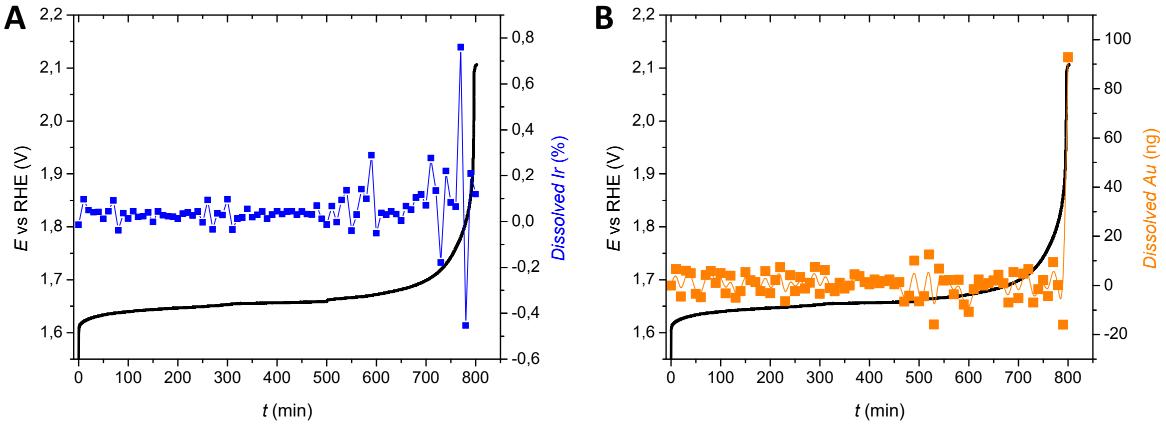

Figure S13 Non-cumulative evolution of Ir and Au dissolution in the course of galvanostatic NP-TF-RDE-based OER using Au as substrate material. (A) ICP-MS-derived loss of Ir during galvanostatic OER (relative to the initial amount of catalyst drop-casted on the Au-RDE electrode). (B) Corresponding Au dissolution from the RDE substrate. The electrolyte analyte was taken every 10 minutes from the electrochemical cell and analyzed postmortem. The concentration of dissolved Ir and Au at any given time was calculated after having subtracted the cumulative metal dissolved prior to it. The Ir dissolution rate reaches a maximum value as the onset of the cutoff potential sets in. After having reached the potential plateau, the catalyst dissolution was non-detectable. The Au dissolution rate markedly rises as soon as the potential reaches the plateau value that follows the cutoff. Applied electrolysis conditions: 50 μg_{Ir} cm^{-2} catalyst loading, j = 10 mA cm^{-2} and f = 1000 rpm in 0.1 M H_2SO_4 supporting electrolyte.

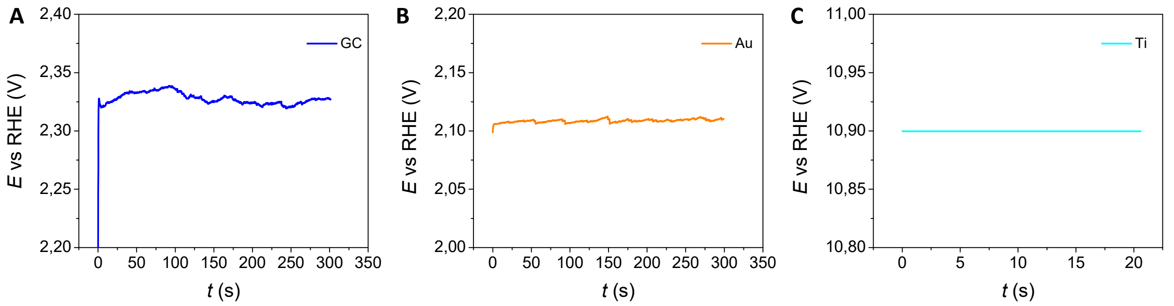

Figure S14 Non-existent OER activity recovery in NP-TF-iRDE OER experiments. (A)-(C) Potential transients acquired on IrO_x-functionalized GC-, Au- and Ti-iRDE electrodes after having sustained galvanostatic OER electrolysis beyond the emergence of the cutoff potential (5 min) and kept in Ar-saturated electrolyte for at least 30 min. The experimental conditions were: 10 μg_{Ir} cm^{-2} catalyst loading, j = 30 mA cm^{-2} and f = 1000 rpm in 0.1 M H_2SO_4 supporting electrolyte.

Supplementary Note 3

The achieved ECD_{IrOx} catalysts on Ti-RDE tips were to a very large extent compact as shown in **Figure S15A** and **Figure 7E** in the main text. However, a minor fraction of the surface exhibited crevices that locally left bare Ti substrate regions uncovered as shown by SEM and EDS analysis in **Figure S15B-D.** Those permeable regions underwent very likely local Ti passivation under the oxidizing applied galvanostatic OER conditions (30 mA cm^{-2} for 20 h). This explains the relatively minor potential increase observed at initial stages of the stability test displayed in **Figure 7F** of the main text. Nevertheless, this ECD_{IrOx} catalyst compactly deposited on Ti-RDE substrates proved to sustain very long stressing tests that allowed actual intrinsic catalyst stability assessment (**Figure 7G-H** in the main text). We suggest that optimized deposition conditions will enable pinhole-free IrO_x catalyst layers with outstanding stabilities that could be transferred to MEA approaches.

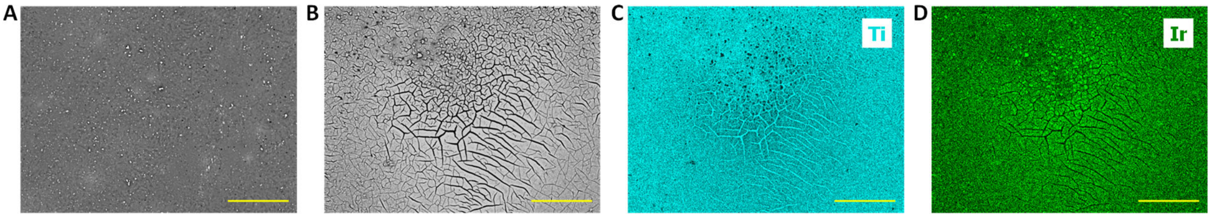

Figure S15 Local discontinuity of ECD_{IrOx} layer on Ti-RDE electrodes. (A) and (B)-(D) show SEM and EDS analysis of a sample region where the ECD_{IrOx} layer was locally disrupted by crevices. Such locations are not representative of the overall continuous catalyst layer. The scale bars represent 25 µm.

References

1. Choe, S.; Lee, B.-S.; Cho, M. K.; Kim, H.-J.; Henkensmeier, D.; Yoo, S. J.; Kim, J. Y.; Lee, S. Y.; Park, H. S.; Jang, J. H., Electrodeposited IrO2/Ti electrodes as durable and cost-effective anodes in high-temperature polymer-membrane-electrolyte water electrolyzers. *Appl. Catal. B: Environ.* **2018,** *226*, 289-294.
2. Zdunek, A. D.; Selman, J. R., A Novel Rotating Disk Electrode Cell Design: The Inverted Rotating Disk Electrode. *J. Electrochem. Soc.* **1992,** *139* (9), 2549.
3. Moreno-García, P.; Kovács, N.; Grozovski, V.; Gálvez-Vázquez, M. d. J.; Vesztergom, S.; Broekmann, P., Toward CO2 Electroreduction under Controlled Mass Flow Conditions: A Combined Inverted RDE and Gas Chromatography Approach. *Anal. Chem.* **2020,** *92* (6), 4301-4308.
4. Liu, Y.; Webb, S.; Moreno-García, P.; Kulkarni, A.; Maroni, P.; Broekmann, P.; Milton, R. D., Facile Functionalization of Carbon Electrodes for Efficient Electroenzymatic Hydrogen Production. *JACS Au* **2023,** *3* (1), 124-130.
5. Moreno-García, P.; Grozovski, V.; Vázquez, M. d. J. G.; Mysuru, N.; Kiran, K.; Kovács, N.; Hou, Y.; Vesztergom, S.; Broekmann, P., Inverted RDE (iRDE) as Novel Test Bed for Studies on Additive-Assisted Metal Deposition under Gas-Evolution Conditions. *J. Electrochem. Soc.* **2020,** *167* (4), 042503.
6. Petzoldt, P. J.; Kwan, J. T. H.; Bonakdarpour, A.; Wilkinson, D. P., Deconvoluting Reversible and Irreversible Degradation Phenomena in OER Catalyst Coated Membranes Using a Modified RDE Technique. *J. Electrochem. Soc.* **2021,** *168* (2), 026507.
7. McCrory, C. C. L.; Jung, S.; Peters, J. C.; Jaramillo, T. F., Benchmarking Heterogeneous Electrocatalysts for the Oxygen Evolution Reaction. *J. Am. Chem. Soc.* **2013,** *135* (45), 16977-16987.
8. El-Sayed, H. A.; Weiß, A.; Olbrich, L. F.; Putro, G. P.; Gasteiger, H. A., OER Catalyst Stability Investigation Using RDE Technique: A Stability Measure or an Artifact? *J. Electrochem. Soc.* **2019,** *166* (8), F458.
9. Trogisch, N.; Koch, M.; El Sawy, E. N.; El-Sayed, H. A., Microscopic Bubble Accumulation: The Missing Factor in Evaluating Oxygen Evolution Catalyst Stability during Accelerated Stress Tests. *ACS Catal.* **2022,** *12* (21), 13715-13724.
10. Hartig-Weiss, A.; Tovini, M. F.; Gasteiger, H. A.; El-Sayed, H. A., OER Catalyst Durability Tests Using the Rotating Disk Electrode Technique: The Reason Why This Leads to Erroneous Conclusions. *ACS Appl. Energy Mater.* **2020,** *3* (11), 10323-10327.
11. Garcia, A. C.; Koper, M. T. M., Effect of Saturating the Electrolyte with Oxygen on the Activity for the Oxygen Evolution Reaction. *ACS Catal.* **2018,** *8* (10), 9359-9363.
12. Brandon, N. P.; Kelsall, G. H.; Levine, S.; Smith, A. L., Interfacial electrical properties of electrogenerated bubbles. *J. Appl. Electrochem.* **1985,** *15* (4), 485-493.
13. Zeradjanin, A. R.; Narangoda, P.; Spanos, I.; Masa, J.; Schlögl, R., How to minimise destabilising effect of gas bubbles on water splitting electrocatalysts? *Curr. Opin. Electrochem.* **2021,** *30*, 100797.
14. Vogt, H., The Concentration Overpotential of Gas Evolving Electrodes as a Multiple Problem of Mass Transfer. *J. Electrochem. Soc.* **1990,** *137* (4), 1179.
15. Zhao, X.; Ren, H.; Luo, L., Gas Bubbles in Electrochemical Gas Evolution Reactions. *Langmuir* **2019,** *35* (16), 5392-5408.
16. Suntivich, J.; May, K. J.; Gasteiger, H. A.; Goodenough, J. B.; Shao-Horn, Y., A Perovskite Oxide Optimized for Oxygen Evolution Catalysis from Molecular Orbital Principles. *Science* **2011,** *334* (6061), 1383-1385.
17. Zheng, Y.-R.; Vernieres, J.; Wang, Z.; Zhang, K.; Hochfilzer, D.; Krempl, K.; Liao, T.-W.; Presel, F.; Altantzis, T.; Fatermans, J.; Scott, S. B.; Secher, N. M.; Moon, C.; Liu, P.; Bals, S.; Van Aert, S.; Cao, A.; Anand, M.; Nørskov, J. K.; Kibsgaard, J.; Chorkendorff, I., Monitoring oxygen production on mass-selected iridium–tantalum oxide electrocatalysts. *Nat. Energy* **2022,** *7* (1), 55-64.

18. Kim, Y.-T.; Lopes, P. P.; Park, S.-A.; Lee, A. Y.; Lim, J.; Lee, H.; Back, S.; Jung, Y.; Danilovic, N.; Stamenkovic, V.; Erlebacher, J.; Snyder, J.; Markovic, N. M., Balancing activity, stability and conductivity of nanoporous core-shell iridium/iridium oxide oxygen evolution catalysts. *Nat. Commun.* **2017,** *8* (1), 1449.
